# Targeting secondary protein complexes in drug discovery: studying the druggability and chemical biology of the HSP70/BAG1 complex†
†Electronic supplementary information (ESI) available. See DOI: 10.1039/c7cc01376k


**DOI:** 10.1039/c7cc01376k

**Published:** 2017-04-21

**Authors:** Lindsay E. Evans, Keith Jones, Matthew D. Cheeseman

**Affiliations:** a Cancer Research UK Cancer Therapeutics Unit at The Institute of Cancer Research , London SW7 3RP , UK . Email: matthew.cheeseman@icr.ac.uk

## Abstract

Proteins typically carry out their biological functions as multi-protein complexes, which can significantly affect the affinity of small-molecule inhibitors. HSP70 is an important target in oncology, so to study its chemical biology and the drug discovery potential of the HSP70/BAG1 complex, we designed a high-affinity non-nucleotide fluorescence polarisation probe.

## Abstract 2

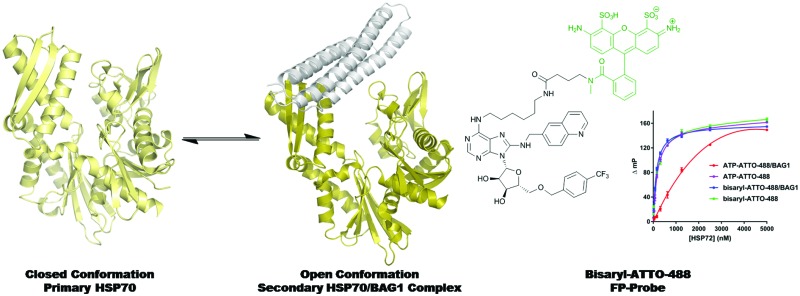
A non-nucleotide FP-probe was designed to study the mechanism of action and druggability of the secondary HSP70/BAG1 complex.

## 

The search for new therapeutics used to rely on cell-based assays to discover bioactive small-molecule hit matter.^1^ Following the advent of readily available recombinant proteins, this phenotypic paradigm was largely replaced by high-throughput screening against individual protein targets.^2^ Although the molecularly-targeted approach has delivered considerable success against certain protein families,^3^ particularly kinases,^4^ others have proven less amenable. This has led to many proteins being considered undruggable, often after a failure to translate biochemical potency to cellular activity.^5^ Proteins carry out their functions through the formation of multi-protein complexes;^6^ however, assays using recombinant proteins generally aim for simplicity in their design, measuring ligand affinity only for individual proteins. The opportunity to compare the druggability of small-molecule binding sites of primary *versus* secondary protein complexes is then lost and optimisation against an isolated target could have limited biological relevance, especially for proteins with a high degree of conformational flexibility.^7^

The 70 kDa heat shock protein family (HSP70) are molecular chaperones responsible for maintaining cell homeostasis^8^ and as such have become an important and popular target in oncology.^9^ The complexity of their catalytic cycle has been well studied^10^ but despite the research efforts of many groups, no drug targeting the HSP70 family and few good chemical tools to investigate their cellular function have been discovered.^11^

Our analysis of the proposed HSP70 catalytic cycle suggested that the protein rarely, if ever, is not in complex with other co-chaperones.^10^ HSP70 carries out its function in an ATP-dependent manner;^10^ the co-chaperone nucleotide-exchange factor (NEF) BAG family molecular chaperone regulator 1 (BAG1) promotes the release of the tight-binding hydrolysis product, ADP/P_i_, allowing ATP to rebind and agonise the catalytic cycle.^12^ Owing to the challenges of targeting HSP70, combined with our broad knowledge of its molecular mechanism of action (MOA) and protein binding partners, we decided to use HSP70 to explore strategies for targeting secondary protein complexes in drug discovery.

The nucleotide-binding domain (NBD) of the HSP70 constitutively active homologue, HSC70, in complex with truncated BAG1 (residues 222–334, tr-BAG1)^11^ has been extensively studied using crystallography, allowing accurate comparison with primary HSP70-NBD structures (Fig. 1).^11^ According to these data, HSP70-NBD binds small molecules in three distinct protein conformations. The ATP-bound HSC70-NBD/tr-BAG1 ternary structure (Fig. 1, grey PDB: ; 3FZF)^11^ demonstrates the most open conformation of the binding cleft. In contrast, the secondary HSP72-NBD structure forms a closed conformation when ADP/P_i_ (Fig. 1, blue, PDB: ; 3ATU)^13^ or certain small molecule inhibitors are bound.^14^ The majority of ligands observed by crystallography appear to bind an intermediate HSP70-NBD conformation (Fig. 2, PDB: ; 4IO8)^15^ and no examples of the open conformation have been observed in the absence of tr-BAG1. These structures suggest that BAG1 has a large and significant effect on the conformation of the adenine-binding pocket, so should also strongly affect the affinity of both nucleotide and non-nucleotide ligands that bind at this site.^16^ However, some experimental evidence suggests that the ability of BAG1 to agonise nucleotide-exchange is actually through disruption of the phosphate-binding pocket of the NBD.^17^ To confirm which BAG1 nucleotide-exchange agonism MOA is correct and to assess whether the secondary HSP70/BAG1 complex represented a more druggable target for screening than the primary HSP70 protein, we hypothesised that an adenine-derived probe could be designed to examine the effect of BAG1 on ATP-competitive small-molecule affinity.

**Fig. 1:**
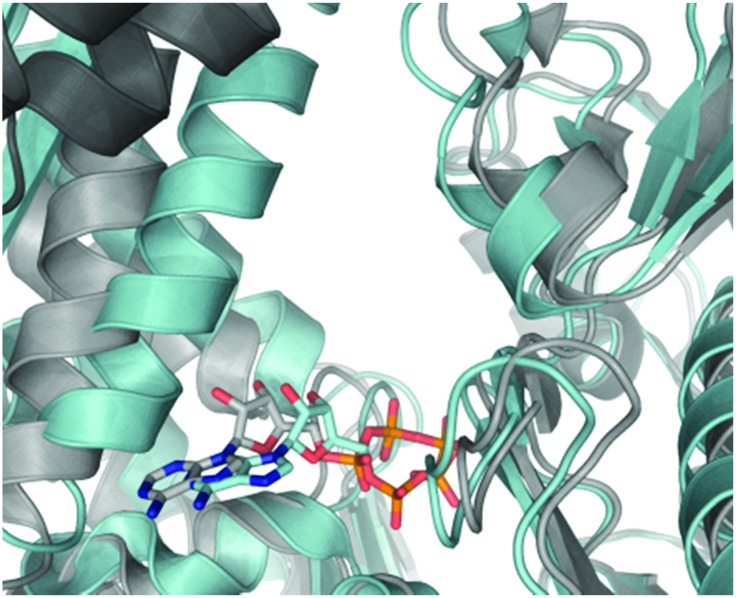
Cartoon overlay (PyMOL Molecular Graphics System, Version 1.8 Schrödinger, LLC) of the open HSC70-NBD/tr-BAG1 ATP-bound complex (HSC70-NBD light grey, tr-BAG1 dark grey, ATP light grey. PDB: 3FZF) with the closed HSP72-NBD ADP/P_i_-bound complex (HSP72-NBD light blue, ADP light blue PDB: ; 3ATU). Blue = nitrogen, red = oxygen, orange = phosphorus, hydrogens, solvent and protein residues omitted for clarity. For a description of key nucleotide interactions see the ESI.†

**Fig. 2:**
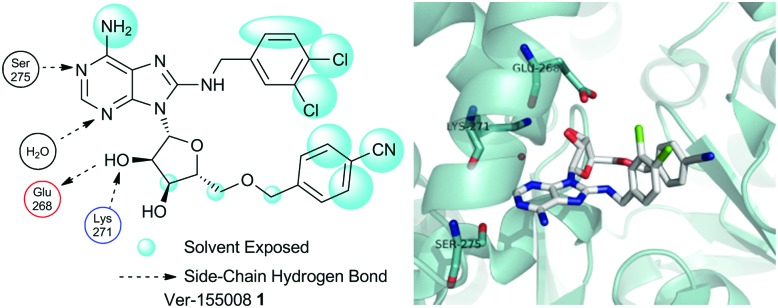
Ver-155008 **1** bound to HSP72-NBD in an intermediate conformation but in the same pocket as the nucleotide ligands (PDB: ; 4IO8), the N6-adenine group is clearly solvent exposed at the front of the pocket (picture adapted from a ligand interaction analysis using MOE 2014.09). Only key residues are shown, solvent and hydrogens are omitted for clarity. Blue = nitrogen, red = oxygen, green = chlorine.

Fluorescence polarisation (FP) is a versatile assay format, which we believed could be exploited for this study.^18^ The assay design required an FP-probe that could bind to both the primary HSP70 protein and the secondary HSP70/BAG1 complex, with high affinity and *via* a binding mode distinct from the nucleotide ligands. The well validated 8-*N*-benzyladenosine HSP70 inhibitors were selected as a start-point for FP-probe design, due to their high affinity for various HSP70 isoforms,^11^,^15^ although their affinity has only ever been determined in the absence of BAG1.

Through analysis of the co-crystal structure of the Ver-155008 **1**^11^ bound to the stress-inducible isoform, heat shock 70 kDa protein 1 (HSP72-NBD, Fig. 2),^16^ we identified the 6-amino position of adenine as being solvent exposed and suitable for linker and fluorophore attachment. To maximize the range of inhibitor potencies that can be resolved in an FP-assay, a tight-binding fluorescent probe is essential.^19^ Bisaryl nucleoside **2**, a tight-binding analogue of Ver-155008 **1**, has a reported affinity for HSP72-NBD of *K*_D_ = 60 nM by surface-plasmon resonance analysis,^11^ so was selected as the basis for design of the FP-probe (Scheme 1).

**Scheme 1:**
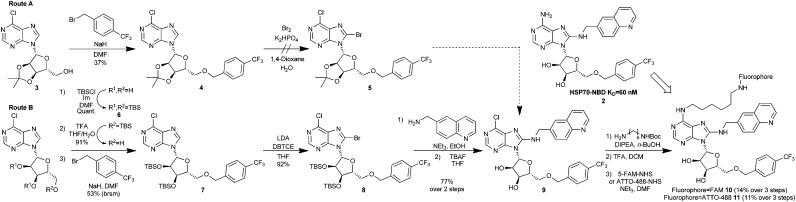
Synthesis of bisaryl FP-probes.

The published route to the bisaryl chemotype^11^ was clearly not be suitable, as key to any successful synthesis would be to introduce the fluorophore in the final step, owing to its instability and difficult purification. Therefore, we designed a route which maintained an electrophilic C6-chloroadenine substituent until the final steps. Starting from the commercially available 3′,4′-acetonide-protected 6-chloroadenosine **3** (Scheme 1, Route A), anionic 5′-*O*-benzylation gave nucleoside **4** in low to moderate yield, due to competing oligomer formation from attack of the anion on the C6-chloro position. Unfortunately, without the 6-amino substituent in place, **4** was apparently too electron-deficient to undergo bromination at the C8-position and failed to afford **5** using standard conditions,^11^ returning a complex mixture of products. To increase the reactivity of the 6-chloroadenine moiety, we attempted to deprotonate the C8-carbon of **4** using LDA and trap the resulting anion with dibromotetrachloroethane (DBTCE), but again without success, as only starting material was recovered. To complete the synthesis of the FP-probe, it proved necessary to change the ribose protecting groups from acetonide to 3′,4′-tributyldimethylsilylethers (TBS) (Scheme 1, Route B). Following selective primary TBS-ether deprotection of the tris-TBS-protected 6-chloroadenosine **6** with a 4 : 1 : 1 mixture of THF/TFA/H_2_O and anionic 5′-*O*-benzylation of the resulting alcohol, treatment of bis-*O*-TBS-6-chloroadenosine **7** with LDA and DBTCE, now gave 8-bromoadenosine **8** in 92% yield. We speculated that the success of this transformation was due to a change in the adenosine conformation caused by the protecting group swap. The aminoquinoline group was then added *via* S_N_Ar reaction, with excellent (>10 : 1) selectivity for C8- over C6-substitution, followed by deprotection of the *O*-TBS-groups to give the key FP-probe precursor, 6-chloroadenosine derivative **9**, in 77% yield. Finally, addition of the diamine linker and *N*-Boc-deprotection was followed by fluorescein-labelling of the primary amine with 5-FAM-NHS-ester to afford the desired FP-probe **10** in 8 steps and 5% overall yield.

The first bisaryl FP-probe **10** we designed used a standard fluorescein fluorophore but displayed an unusually high degree of background polarisation in the absence of protein, giving a small assay window (∼30 mP, Fig. 3A). As a result, the assay was not statistically robust, so could not be used in competition experiments (*Z*′ = 0.36).^11^,^20^,^21^ We hypothesised that the high lipophilicity of **10** (clog *P* = 6.9) was causing the small binding window, either due to aggregation, even at very low concentrations, or its large hydrate volume.^22^ Because the lipophilicity of the specific-ligand portion of the probe was fixed by the need for high affinity to the HSP70 target, we focused on changes to the solvent exposed fluorophore. ATTO-488, a green-shifted fluorophore that contains two sulfonic acid groups, was selected as an alternative to fluorescein (see ESI†), as this reduced the clog *P* of the bisaryl probe **11** to –3.9.^21^ The binding window of ATTO-488 bisaryl FP-probe **11** measured at a fixed and apparently saturating concentration of HSP72 (5 μM), was now greater than 150 mP (*Z*′ = 0.63, Fig. 3A and Fig. S1, ESI†).

**Fig. 3:**
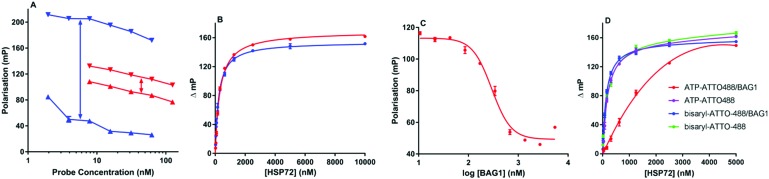
HSP72 fluorescence polarisation assays. All points are tested in triplicate and are represented as the arithmetic mean ± SEM. All graphs were prepared and analysed using Graphpad Prism 7.01. (A) Polarization values (mP) for 2–63 nM bisaryl-ATTO-488 FP-probe **11** (blue) and 8–125 nM bisaryl-FAM **10** (red) in the presence () and absence (▲) of 5.0 μM HSP72, ↔ represents the maximum potential assay window. (B) Representative binding isotherms for bisaryl-ATTO-488 FP-probe **11** (blue) and ATP-ATTO-488 (red) with 20 nM to 10 μM HSP72 fitted to a one-site specific binding model. (C) Displacement curve for ATP-ATTO-488 binding to HSP72 in the presence of 10 nM to 5.5 μM BAG1, isolated BAG1 displayed no measurable affinity for ATP-ATTO-488 or bisaryl-ATTO-488 **11** see ESI.† (D) Affinity of bisaryl-ATTO-488 **11** for HSP72 is maintained in the presence of a saturating concentration of BAG1 (700 nM), whilst the ATP-ATTO-488 probe is clearly reduced.

To determine the binding affinity of bisaryl FP-probe **11** for HSP72, increasing concentrations of the protein were titrated against a fixed concentration (10 nM) of the FP-probe. Commercially available ATP-ATTO-488 was originally used as a positive control (Fig. 3B); however, it became clear that the affinity of this nucleotide-derived probe was time-dependent (Fig. S2, ESI†), initially returning an apparent *K*_D_ of only ∼1.5 μM and plateauing at 290 nM (p*K*_D_ = 6.54 ± 0.03, *n* = 4) after 6 hours incubation. We speculated that this decrease in apparent *K*_D_ was due to the slow hydrolysis of the ATP-probe to ADP-ATTO-488 + P_i_. Although HSP72 has low intrinsic ATPase activity,^8^ the high enzyme concentration could catalyse hydrolysis over the time-frame of the assay, with the initial *K*_D_ values reflective of the known modest affinity of ATP for HSP70, whilst the final *K*_D_ value was consistent with the higher affinity ADP ligand.^14^ The bisaryl-ATTO-488 FP-probe **11** displayed no apparent time-dependency in its affinity and subsequent analysis of its binding isotherms to HSP72 revealed a *K*_D_ value of 194 nM (p*K*_D_ = 6.72 ± 0.04, *n* = 4, Fig. 3B). No detergent effects were displayed by either FP-probe (Fig. S3, ESI†) and binding specificity for **11** was confirmed by competition with the parent bisaryladenosine inhibitor **2**, displaying an IC_50_ = 137 nM (pIC_50_ = 6.87 ± 0.05, *n* = 3) at the apparent tight-binding limit for the assay (Fig. S4, ESI†).^20^ Truncated HSP72-NBD (residues 3 to 382) and HSC70-NBD (residues 4 to 381), often used in crystallography experiments and in the previous affinity assessments with this chemotype,^11^,^15^ displayed higher affinity for the FP-probe **11** than full-length HSP72 (Fig. S5, ESI†).

To investigate the role of the secondary protein complex in the binding affinity of ligands for HSP70, we needed to confirm the formation of the protein–protein interaction between HSP72 and BAG1 in solution under the assay conditions. Because BAG1 is a known NEF, we hypothesized that it would reduce the affinity of the ATP-ATTO-488 FP-probe when the secondary complex forms, leading to a reduction in observed polarisation. BAG1 was titrated against a fixed concentration of HSP72 (195 nM) and ATP-ATTO-488 (10 nM), concentrations were selected to give a 50% bound fraction of the FP-probe in the assay.^20^ The increasing concentration of BAG1 clearly reduced the affinity of the ATP-ATTO-488 FP-probe in a dose-dependent and saturatable manner, consistent with the formation of the secondary HSP72/BAG1 protein complex, and reaching a plateau at 680 nM BAG1 and 50 mP (Fig. 3C). These data are consistent with previous studies, suggesting that the secondary HSP70/BAG1 complex displays much lower affinity for nucleotide ligands but the binding of BAG1 and the nucleotide is not necessarily mutually exclusive.^10^,^17^,^18^

To assess what effect the secondary HSP72/BAG1 complex had on the apparent affinity of the non-nucleotide bisaryl-ATTO-488 FP-probe **11**, we carried out a similar BAG1 study. In stark contrast to the ATP derived FP-probe, BAG1 treatment at an apparently saturating concentration (700 nM) against the HSP72/bisaryl-ATTO-488 **11** complex, resulted in no significant change in affinity of the probe (Fig. 3D). No interaction between BAG1 and either ATP-ATTO-488 or bisaryl-ATTO-488 **11** was seen in the absence of HSP72 (Fig. S6, ESI†). To confirm that the binding of BAG1 to HSP72 was not mutually exclusive, we screened the nucleotides ADP and ATP in a competition assay with bisaryl-ATTO-488 **11** and HSP72 at a 50% bound fraction, with and without a saturating concentration of BAG1 (700 nM) (Fig. S7, ESI†). ADP (Table 1, entry 1) displayed a significant 6.8-fold decrease in its affinity (IC_50_ = 2.2 μM, pIC_50_ = 5.66 ± 0.04, *n* = 3) for the secondary complex compared to the primary HSP72 protein, with ATP (Table 1, entry 2) displaying similar results. These data confirm the formation of the HSP72/BAG1/bisaryl-ATTO-488 **11** ternary complex under the assay conditions and the weaker affinity of nucleotides for the secondary complex. The known non-nucleotide ligands, quinoline **12** (Table 1, entry 3) and sangivamycin **13** (Table 1, entry 4), displayed no significant change in their affinity for the HSP72/BAG1 complex compared to the primary protein, despite exploiting many of the same interactions in the adenine-binding pocket as the nucleotide ligands (see Fig. S8, ESI†).^15^

**Table 1:** Displacement of bisaryl-ATTO-488 **11** FP-probe from the primary HSP72 protein and secondary HSP72/BAG1 complex using nucleotide and non-nucleotide ligands

Entry	Compd	IC_50_^*a*^ (μM)	
		–BAG1	+BAG1^*b*^
1	ADP	0.33	2.2
2	ATP	0.65	>10
3	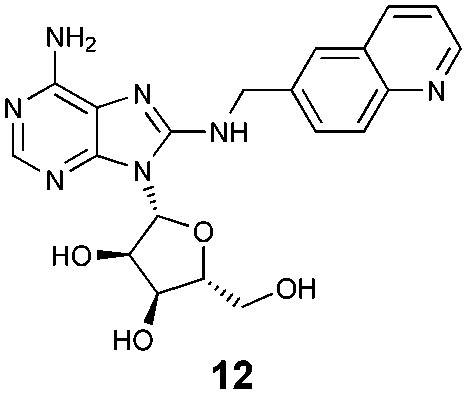	2.0	2.0
4	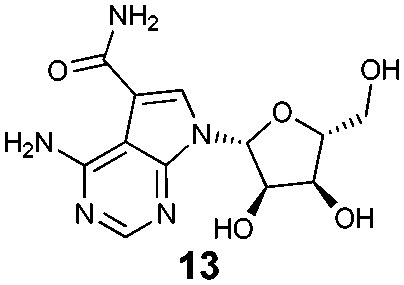	2.4	1.0

^*a*^Geometric mean of at least 3 independent experiments.

^*b*^700 nM BAG1 or the equivalent volume of the BAG1 buffer, was plated with 140 nM HSP72 (or 180 nM for the plates without BAG1) to give a 50% bound fraction and were incubated for 16 h prior to being read.

By developing an FP-probe derived from a high affinity non-nucleotide ligand, we were able to investigate the role of the NEF BAG1 in its secondary complex with HSP70. Our data suggests that BAG1 agonises nucleotide-exchange through conformational changes of the phosphate-binding pocket rather than the adenosine-binding pocket, as had been suggested by crystallography; while non-nucleotide ligands of HSP70 are apparently purely non-competitive with BAG1. This confirms the mechanistic hypothesis that phosphate dissociation is rate-determining in the NEF agonism of HSP70. Future crystallography efforts of the secondary complex should focus on utilizing full-length BAG1 to give greater insight into the molecular MOA, as tr-BAG1 cannot interact with the phosphate-binding pocket. Whether the new and unexplored phosphate-binding pocket conformation of the secondary HSP70/BAG1 complexes can be exploited to discover new hit-matter for inhibitors of HSP70 is currently unclear, as there are no known small-molecule non-nucleotide ligands that bind there. A screen using the bisaryl-ATTO-488 **11** FP-probe to confirm whether the HSP70/BAG1 complex can generate novel hit-matter is currently under investigation.

This work was financially supported by Wellcome Trust studentship, (WEL075) http://www.wellcome.ac.uk/ (LEE) and Cancer Research UK, C309/A8274 and C309/A11566 ; www.cancerresearchuk.org/ (KJ, MDC). We would like to thank Norhakim Yahya for help with protein production.

## Supplementary Material

Supplementary information
