# Supplementary material for: Targeting secondary protein complexes in drug discovery: studying the druggability and chemical biology of the HSP70/BAG1 complex
Source: Chem Commun (Camb). 2017 Apr 21;53(37):5167–70. doi: 10.1039/c7cc01376k (PMC5708526; doi:10.1039/c7cc01376k)
Supplement: Supplementary file 1 [file CC-053-C7CC01376K-s001.pdf]

Electronic Supplementary Material (ESI) for ChemComm.
This journal is © The Royal Society of Chemistry 2017

Electronic Supplementary Information

Targeting Secondary Protein Complexes in Drug Discovery: Studying the Druggability and Chemical Biology of the HSP70/BAG1 Complex

Lindsay E. Evans,[a] *Keith Jones*[a] *and Matthew D. Cheeseman,*[a]

[a]Cancer Research UK Cancer Therapeutics Unit at The Institute of Cancer Research, London SW7 3RP, United Kingdom

Contents:

Biochemical Experimental S2-S6

Chemistry Experimental S7-S15

NMR Spectra of Key Compounds S16-S19

HSP70 Crystallography Analysis S20-S22

Biochemical Experimental

Abbreviations

CHAPS	(3-((3-cholamidopropyl) dimethylammonio)-1-propanesulfonate)
DTT	Dithiothreitol
TRIS	Tris(hydroxymethyl)aminomethane

Material and Methods

General Information: Unless otherwise stated the aqueous assay buffer contained 50 mM TRIS base pH 7.4, 150 mM NaCl, 6 mM $MgCl_2$, 1 mM DTT, 0.1% (wt/wt) CHAPS. The assay was conducted using 384 Plus F ProxiPlates (PerkinElmer) with a final assay volume of 10 µL. Plates were centrifuged at 1000 rpm for 1 minute prior to incubation and read using an 2103 Envision Multilable Plate Reader. Excitation and emission wavelengths used for green probes were 480 nm and 535 nm, respectively. Fluorescence polarisation was measured in units of millipolarization (mP). All experiments were performed in triplicate, unless otherwise stated. Data were plotted and analysed using GraphPad Prism 6, graphical data represents the mean ± standard error of the mean for a single representative experiment. ATP-derived fluorescent probes (1.0 mM in aqueous) were purchased from Jena Bioscience. Human BAG-1M (10.9 µM in 20 mM TRIS base pH 7.4, 100 mM NaCl, 1 mM DTT, 1% (v/v) glycerol) was purchased from Enzo Life Sciences.

Determination of optimal probe concentration: To each well, 5 µL of HSP72 (10 µM in assay buffer) or 5 µL assay buffer and increasing concentrations of probe (5 µL, two-fold dilution series) were added. Fluorescence polarisation values for the tracer ± protein were plotted using GraphPad prism 6.

Fluorescent probe K_D determination: To each well, 5 µL of probe molecule (20 nM in assay buffer) and increasing concentrations of HSP70 protein (5 µL, two-fold dilution series) were added. Fluorescence polarisation values for tracer control wells (10 nM probe in assay buffer only) were subtracted from each data point prior to data analysis. K_D determination was performed using non-linear regression analysis (GraphPad Prism 6, one site–specific binding model).

Z' determination: To each well, 5 µL of probe (20 nM in assay buffer) and either 5 µL of HSP70 (in assay buffer) or 5 µL of assay buffer were added. Fluorescent polarisation values from 96 wells were used to calculate the mean and standard deviation for each data set. Z'-factors were calculated using the equation given below (sample refers to probe in the absence of protein and control refers to probe in the presence of protein).

$$Z' = 1 - \frac{(3\sigma_{positive} + 3\sigma_{negative})}{|\mu_{positive} - \mu_{negative}|}$$

Compound IC_{50} determination: Compounds (0.2 µL at 50 x screening concentration in DMSO) were dispensed using an ECHO 550 Liquid Handler (Labcyte Inc.). To the corresponding wells was added, 5 µL of probe molecule (20 nM in assay buffer) and 5 µL of protein (two times their final concentration in assay buffer) to give a 50% bound fraction. Tracer controls (10 nM probe molecule only) and bound tracer controls (10 nM probe in the presence of appropriate protein concentration) were included on each assay plate. IC_{50} determination was performed using non-linear least squares curve fitting (GraphPad Prism 6, log(inhibitor) vs. response—variable slope (four parameters)).

BAG-1 titration: To each well, ATP-ATTO488 and HSP70 at two times their final concentration (5 µL in assay buffer) was added and the plate incubated in the dark for 16 hours. Increasing concentrations of BAG1 protein (5 µL, two-fold dilution series in 20

mM TRIS base pH 7.4, 100 mM NaCl, 1 mM DTT, 1% (v/v) glycerol) or BAG1 replacement buffer (5 µL, 20 mM TRIS base pH 7.4, 100 mM NaCl, 1 mM DTT, 1% (v/v) glycerol) was added to the corresponding wells of the assay plate. Fluorescence polarisation values for probe and HSP72 ± BAG1 were plotted using GraphPad prism 6.

K_D determination with BAG1: To each well, 5 µL containing probe molecule (20 nM in assay buffer) and BAG1 protein (1.4 µM in assay buffer) and increasing concentrations of HSP70 protein (5 µL, two-fold dilution series) were added. Fluorescence polarisation values for tracer control wells (10 nM probe in assay buffer only) were subtracted from each data point prior to data analysis. K_D determination was performed using non-linear regression analysis (GraphPad Prism 6, one site–specific binding model).

Compound screen with and without BAG-1: Compounds (0.2 µL at 50 x screening concentration in DMSO) were dispensed using an ECHO 550 Liquid Handler (Labcyte Inc.). To the corresponding wells was added, 5 µL of probe molecule (20 nM in assay buffer), 2.5 µL of HSP72 (four times final concentration in assay buffer) and either 2.5 µL of BAG-1 (2.8 µM in 20 mM TRIS base pH 7.4, 100 mM NaCl, 1 mM DTT, 1% (v/v) glycerol) or 2.5 µL of BAG1 replacement buffer (20 mM TRIS base pH 7.4, 100 mM NaCl, 1 mM DTT, 1% (v/v) glycerol). The assay was performed in duplicate. Tracer controls (10 nM probe molecule only) and bound tracer controls (10 nM probe in the presence of appropriate protein concentration) were included on each assay plate. IC_{50} determination was performed using non-linear least squares curve fitting (GraphPad Prism 6, log(inhibitor) vs. response—variable slope (four parameters)).

Figures S1 – S7

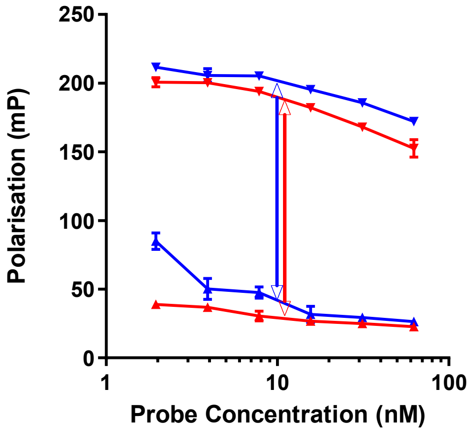

Fig. S1. Determination of assay binding window for bisaryl-ATTO-488 and ATP-ATTO-488. Polarisation values (mP) for 2-63 nM ATP-ATTO-488 (red) and bisaryl ATTO-488 (blue) in the presence (▼) and absence (▲) of 5 µM HSP72, ↔ represents the assay window at 10 nM probe concentration. All data points were tested in triplicate and are represented as the arithmetic mean ± SEM.

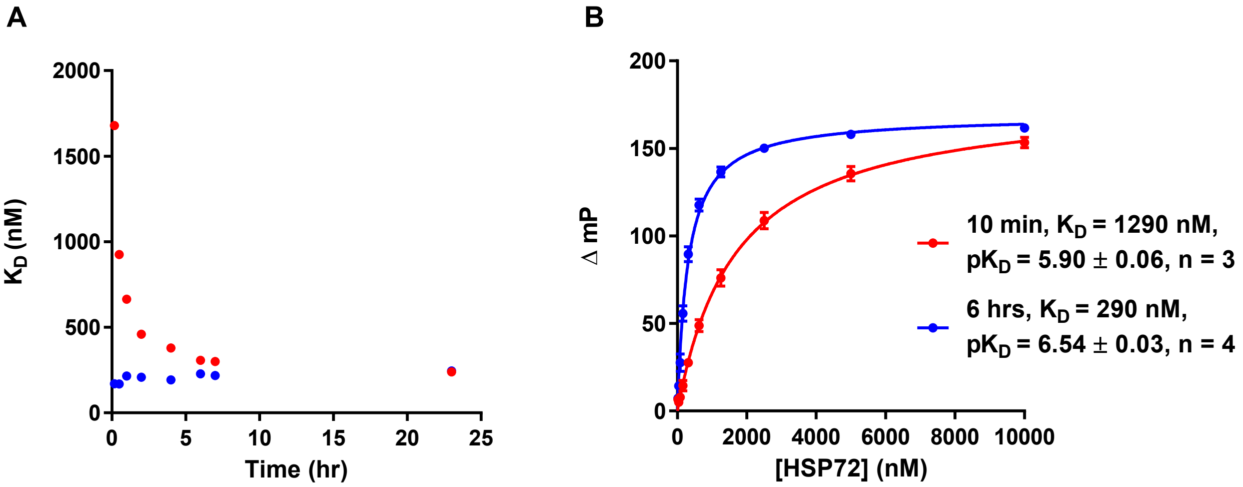

Fig. S2. Time dependence of affinity of ATP-ATTO-488 binding to HSP72. a) Plot of K_D vs. time for ATP-ATTO-488 (red) and bisaryl-ATTO-488 (blue) binding to HSP72; b) Representative binding isotherms for ATP-ATTO-488 with 20 nM to 10 µM HSP72 at 10 minutes incubation (red) and 6 hours incubation (blue). K_D determination was performed using non-linear regression analysis (one site–specific binding model), all data points were tested in triplicate and are represented as the arithmetic mean ± SEM. pK_D=-log K_D (M). pK_D values are quoted as the geometric mean±SEM of *n* independent repeats.

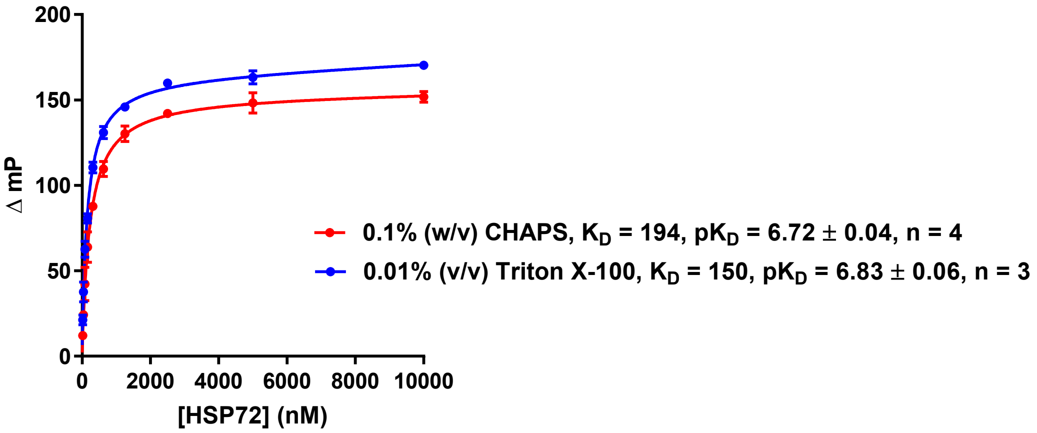

Fig. S3. Bisaryl-ATTO-488 binding to HSP72 is not detergent dependent. Representative binding isotherms for bisaryl-ATTO-488 binding to HSP72 in the presence of 0.1% (w/v) CHAPS (red) or 0.01% Triton X-100 (v/v) (blue) detergent. K_D determination was performed using non-linear regression analysis (one site–specific binding model), all data points were tested in triplicate and were represented as the arithmetic mean ± SEM. pK_D=-log K_D (M). pK_D values are quoted as the geometric mean±SEM of *n* independent repeats.

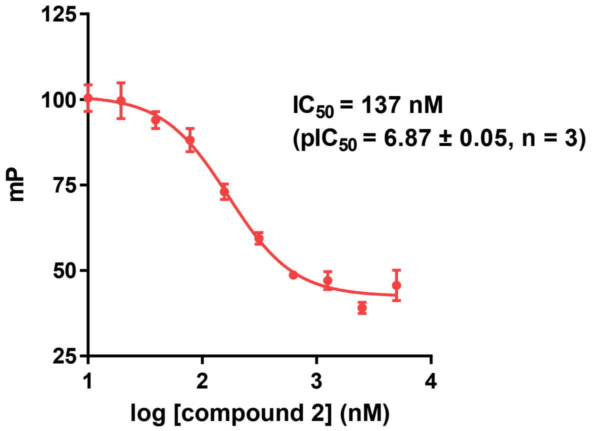

Fig. S4. Competitive displacement curve for bisarryl-ATTO-448 and HSP72 with compound 2. IC_{50} determination was performed using non-linear least squares curve fitting (GraphPad Prism 6, log(inhibitor) vs. response—variable slope (four parameters)), all data points were tested in triplicate and are represented as the arithmetic mean ± SEM. pK_D=-log K_D (M). pK_D values are quoted as the geometric mean±SEM of *n* independent repeats.

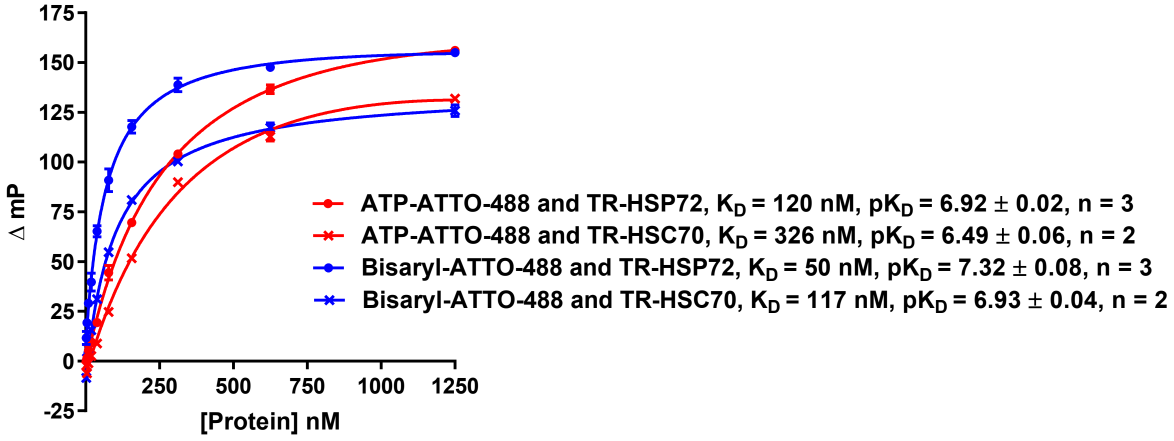

Fig. S5. Bisaryl-ATTO-488 and ATP-ATTO-488 binding to truncated HSP72 and HSC70. Representative binding isotherms for bisaryl-ATTO-488 (blue) and ATP-ATTO-488 (red) binding to TR-HSP72 (aa 3-382) (•) and TR-HSC70 (aa 4-381) (**x**). K_D determination was performed using non-linear regression analysis (one site–specific binding model), all data points were tested in triplicate and are represented as the arithmetic mean ± SEM. pK_D=-log K_D (M). pK_D values are quoted as the geometric mean±SEM of *n* independent repeats.

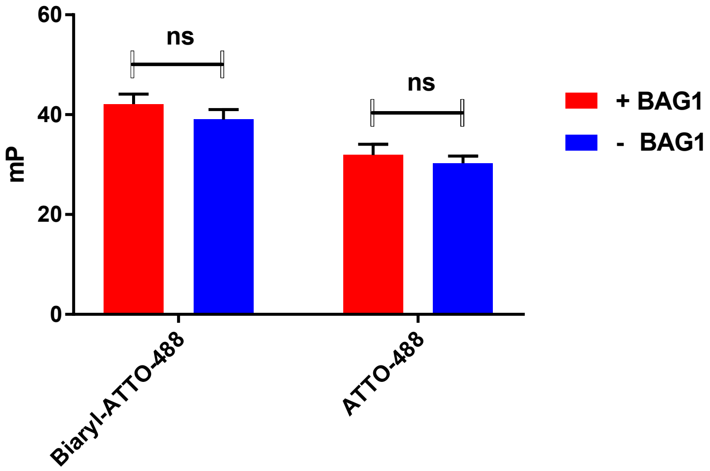

Fig. S6. BAG1 does not interact with bisaryl-ATTO-488 or ATP-ATTO-488 in the absence of HSP72. Polarisation values (mP) for 10 nM bisaryl-ATTO-488 and 10 nM ATP-ATTO-488 in the presence (red) or absence (blue) of 1 µM BAG1. All data points were tested in triplicate and are represented as the arithmetic mean ± SEM, ns = not significant (p>0.05, Student's t-test).

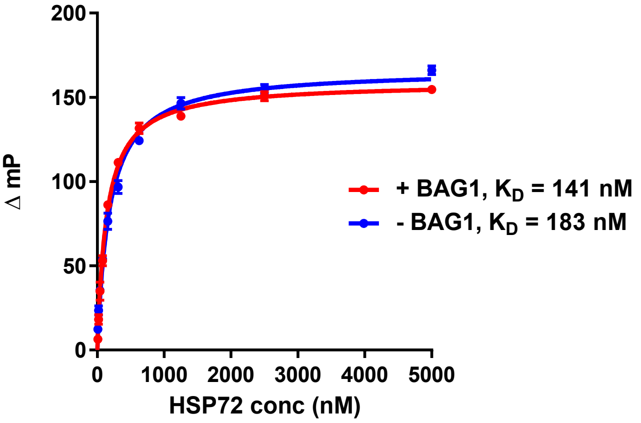

Fig. S7. Affinity of bisaryl-ATTO-488 for HSP72 is maintained in the presence of BAG1. Binding isotherm for biaryl-ATTO-488 with 700 nM BAG1 (blue) or without BAG1 (red). K_D determination was performed using non-linear regression analysis (one site–specific binding model), all data points were tested in triplicate and are represented as the arithmetic mean ± SEM.

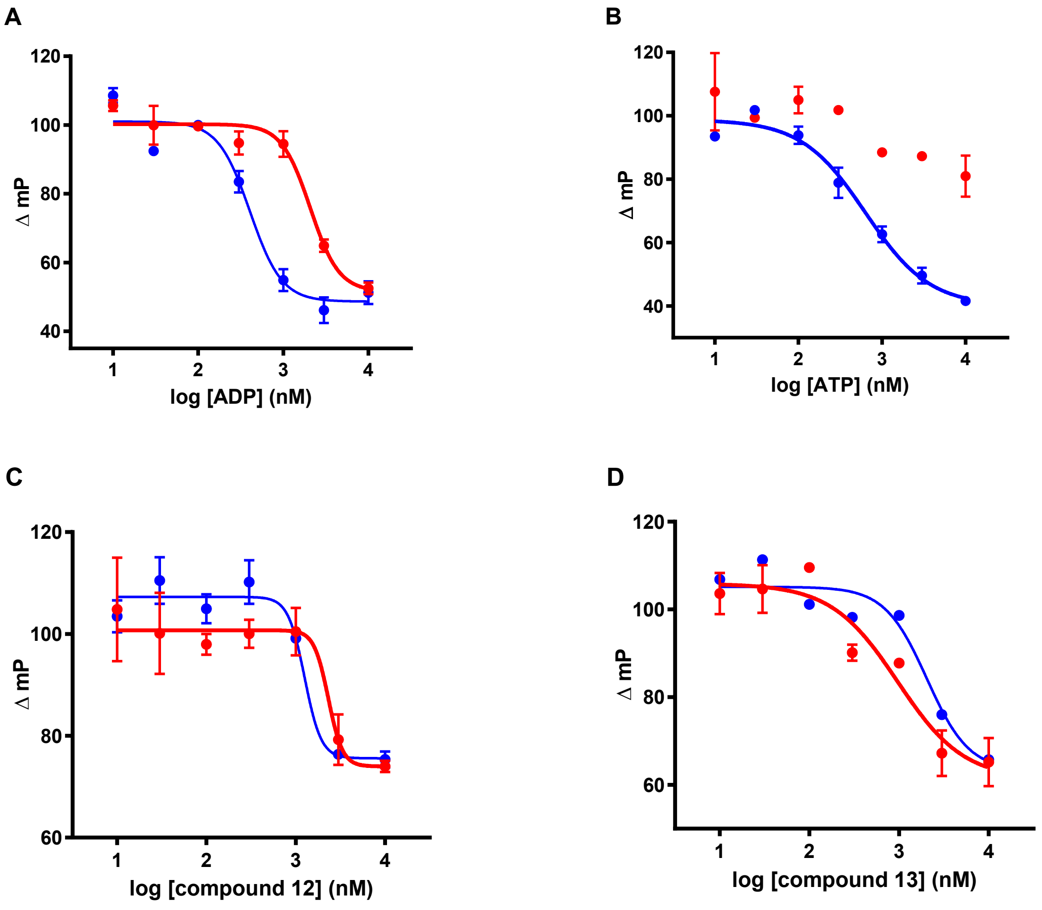

Fig. S8. Representative competitive binding curves for ADP (A), ATP (B), compound 12 (C) and compound 13 (D) in the presence and absence of BAG1. Competition experiments in the presence of BAG1 (red) were performed using 10 nM biaryl-ATTO-488, 700 nM BAG1 and 140 nM HSP70 to give a 50% bound fraction. Competition experiments in the absence of BAG1 (blue) were performed using 10 nM biaryl-ATTO-488 and 180 nM HSP70 to give a 50% bound fraction. IC_{50} determination was performed using non-linear regression analysis (log(inhibitor) vs. response - Variable slope (four parameters)), all data points were tested in duplicate and are represented as the arithmetic mean ± SEM.

Chemistry Experimental

General experimental: Reagents and solvents were purchased from commercial suppliers (Acros, Alfa Aesar, Apollo, Cambridge peptides, Fisher scientific, Fluorochem, Lumiprobe, Manchester Organics, Sigma-Aldrich, Thermo Scientific and VWR) and used without further purification.

Analytical thin layer chromatography (TLC) was performed on pre-coated aluminium sheets (60 F245 nm, Merck) and visualised by short-wave UV light and potassium permanganate dips. Flash column chromatography was carried out using Merck silica gel 60 (40-65 µm).

Purification by semi-preparative HPLC was carried out using one of the following sets of conditions:

Method A) Chromatographic separation was carried out at room temperature using a Gilson GX-281 Liquid Handler system combined with a Gilson 322 HPLC pump (Gilson, Middleton, USA) over a 15 minute gradient elution (Grad15mins20mls.m) from 10:90 to 100:0 methanol:water (both modified with 0.1% formic acid) at a flow rate of 20 mL/min. UV-Vis spectra were acquired at 254 nm on a Gilson 156 UV-Vis detector (Gilson, Middleton, USA). Collection was triggered by UV signal, and collected using a Gilson GX-281 Liquid Handler system (Gilson, Middleton, USA).

Method C) Chromatographic separation was carried out at room temperature using a 1200 Series Preparative HPLC (Agilent, Santa Clara, USA) with a Phenomenex Gemini column (5 µm, 250 x 10 mm, C18, Phenomenex, Torrance, USA) using a 15 minute gradient elution (Grad15min.m) from 10:90 to 100:0 methanol:water (both modified with 0.1% formic acid) at a flow rate of 5.0 mL/min. UV-Vis spectra were acquired at 254nm and 280nm on a 1200 Series Prep Scale diode array detector (Agilent, Santa Clara, USA). Collection was triggered by UV and collected on a 1200 Series Fraction Collector (Agilent, Santa Clara, USA).

^{1}H NMR spectra were recorded on a Bruker AMX500 (500 MHz) spectrometer using an internal deuterium lock. Chemical shifts were measured in parts per million (ppm) relative to tetramethylsilane (δ = 0) using the following residual solvent signals: $CDCl_3$ (δ_H 7.26), CD_3OD (δ_H 3.32), and $(CD_3)_2SO$ (δ_H 2.50). Multiplicities are recorded as singlet (s), doublet (d), triplet (t), quartet (q) and multiplet (m), doublet of doublets (dd), doublet of doublet of doublets (ddd), apparent (app), obscured (obs) and broad (br). Coupling constants, J, are measured to the nearest 0.1 Hz. ^{13}C NMR spectra were recorded at 126 MHz on a Bruker Avance 500 MHz spectrometer using an internal deuterium lock. Chemical shifts were measured in parts per million (ppm) relative to tetramethylsilane (δ = 0) using the following residual solvent signals: $CHCl_3$ (δ_C 77.16), CD_3OD (δc 49.00) and $(CD_3)_2SO$ (δ_C 39.52). Chemical shifts are quoted to 0.1 ppm, unless greater accuracy is required. * Denotes peaks observed only in HSQC spectra.

High resolution mass spectrometry was performed on an Agilent 1200 series HPLC and diode array detector coupled to a 6210 time of flight mass spectrometer with dual multimode APCI/ESI source. Analytical separation was carried out at 30 °C on a Merck Chromolith SpeedROD column (RP-18e, 50 x 4.6 mm) or a Merck Purospher STAR column (RP-18e, 30 x 4 mm) using a flow rate of 1.5 mL/min in a 4 minute gradient elution; solvents – aqueous (0.1% formic acid) and methanol. UV Detection was at 254 nm. LC/MS analysis was performed on a Waters Alliance 2795 separations module and a Waters 2487 dual wavelength absorbance detector coupled to a Waters/Micromass LCT time of flight mass spectrometer with ESI source. Analytical separation was carried out at 30 ^{o}C on a Merck Purospher STAR column (RP-18e, 30 x 4 mm) using a flow rate of 1.5 mL/min in a 4 minute gradient elution with detection at 254 nm.

6-Chloro-9-((3a*R*,4*R*,6*R*,6a*R*)-2,2-dimethyl-6-(((4-(trifluoromethyl) benzyl)oxy)methyl)tetrahydrofuro[3,4-*d*][1,3]dioxol-4-yl)-9*H*-purine (Compound 4)

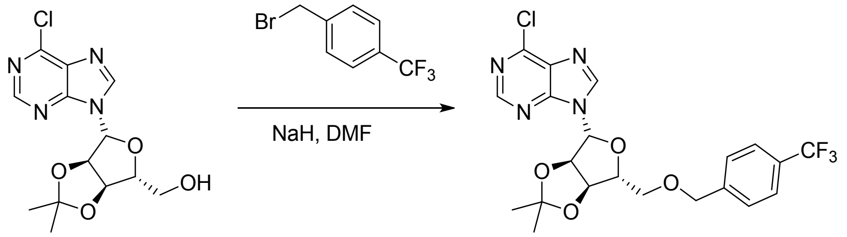

4

Compound **4** was prepared by modification of a literature procedure.[1] 1-(Bromomethyl)-4-(trifluoromethyl)benzene (0.25 g, 1.04 mmol) was added to a stirred solution of ((3a*R*,4*R*,6*R*,6a*R*)-6-(6-chloro-9*H*-purin-9-yl)-2,2-dimethyltetrahydrofuro[3,4-*d*][1,3]dioxol-4-yl)methanol (0.20 g, 0.61 mmol) in DMF (8 mL). After 5 minutes, NaH (60% dispersion in oil, 27 mg, 0.67 mmol) was added and the reaction stirred for 4 hours at room temperature. The reaction mixture was cooled to 0 °C and quenched by the addition of water and diluted with brine. The resulting mixture was extracted with EtOAc and the organic extracts combined, washed with brine and dried over $MgSO_4$. The solvent was removed under reduced pressure and the crude product purified by column chromatography (3:2 cyclohexane/EtOAc) to give compound **4** as a colourless oil (0.11 g, 37%); δ_H (500 MHz, CD_3OD) 8.63 (s, 1H), 8.62 (s, 1H), 7.45 (d, *J* = 8.0 Hz, 2H), 7.22 (d, *J* = 8.0 Hz, 2H), 6.26 (d, *J* = 2.0 Hz, 1H), 5.53 (dd, *J* = 6.0, 2.0 Hz, 1H), 5.09 (dd, *J* = 6.0, 2.0 Hz, 1H), 4.62 (m, 1H), 4.48 (d, *J* = 12.3 Hz, 1H), 4.40 (d, *J* = 12.3 Hz, 1H), 3.79 (dd, *J* = 10.7, 3.0 Hz, 1H), 3.70 (dd, *J* = 10.7, 3.9 Hz, 1H), 1.59 (s, 3H), 1.41 (s, 3H); δ_C (126 MHz, CD_3OD) 152.8, 152.3, 151.2, 146.6, 143.3, 132.8, 130.7 (q, *J* = 32.2 Hz), 128.7, 126.1 (q, *J* = 3.8 Hz), 125.5 (q, *J* = 271.7 Hz), 114.9, 94.3, 88.3, 86.1, 83.5, 73.3, 71.9, 27.4, 25.4; HRMS (ESI) $C_{21}H_{21}ClF_3N_4O_4$ ($M+H^+$) requires 485.1198, found 485.1194.

9-((2*R*,3*R*,4*R*,5*R*)-3,4-Bis((tert-butyldimethylsilyl)oxy)-5-(((tert-butyldimethylsilyl)oxy)methyl)tetrahydrofuran-2-yl)-6-chloro-9*H*-purine (Compound 6)

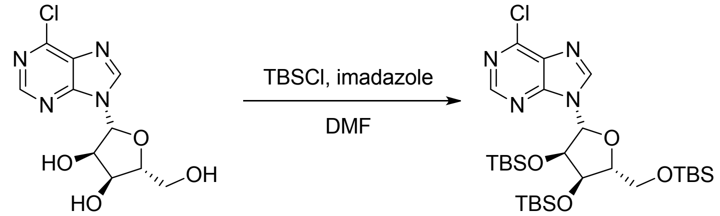

6

Compound **6** was prepared by modification of a literature procedure.[2] (2*R*,3*R*,4*S*,5*R*)-2-(6-chloro-9*H*-purin-9-yl)-5-(hydroxymethyl)tetrahydrofuran-3,4-diol (0.46 g, 1.61 mmol), TBSCl (1.45 g, 9.63 mmol) and imidazole (0.77 g, 11.23 mmol) were dissolved in DMF (17 mL) and the reaction stirred at room temperature for 16 hours. The reaction mixture was then diluted with EtOAc and saturated aqueous Na_2CO_3 solution and the aqueous layer separated and extracted with EtOAc. The

[1] Williamson, D. S.; Borgognoni, J.; Clay, A.; Daniels, Z.; Dokurno, P.; Drysdale, M. J.; Foloppe, N.; Francis, G. L.; Graham, C. J.; Howes, R.; Macias, A. T.; Murray, J. B.; Parsons, R.; Shaw, T.; Surgenor, A. E.; Terry, L.; Wang, Y.; Wood, M.; Massey, A. J. *J. Med. Chem.* **2009**, *52*, 1510.

[2] Kato, K.; Hayakawa, H.; Tanaka, H.; Kumamoto, H.; Shindoh, S.; Shuto, S.; Miyasaka, T. *J. Org. Chem.* **1997**, *62*, 6833.

organic layers were combined, washed with brine and dried over $MgSO_4$. The solvent was partially evaporated under reduced pressure and the crude product recrystallized from EtOAc/water to give compound **6** as a white solid (0.93 g, quantitative yield); mp 133.6–136.0 °C (lit., 138 °C)[2]; δ_H (500 MHz, $CDCl_3$) 8.74 (s, 1H), 8.55 (s, 1H), 6.13 (d, *J* = 5.0 Hz, 1H), 4.59 (app t, *J* = 4.6 Hz, 1H), 4.31 (app t, *J* = 4.0 Hz, 1H), 4.16 (app q, *J* = 3.5 Hz, 1H), 4.02 (dd, *J* = 11.5, 3.5 Hz, 1H), 3.81 (dd, *J* = 11.5, 2.4 Hz, 1H), 0.96 (s, 9H), 0.93 (s, 9H), 0.79 (s, 9H), 0.16 (s, 3H), 0.15 (s, 3H), 0.103 (s, 3H), 0.098 (s, 3H), -0.03 (s, 3H), -0.24 (s, 3H); δ_C (126 MHz, $CDCl_3$) 152.1, 151.7, 151.2, 144.2, 132.2, 88.7, 85.9, 76.6, 72.0, 62.6, 26.3, 26.0, 25.8, 18.7, 18.2, 18.0, -4.2, -4.5, -4.6, -4.9, -5.15, -5.19; HRMS (ESI) $C_{28}H_{54}ClN_4O_4Si_3$ ($M+H^+$) requires 629.3136, found 629.3130.

((2*R*,3*R*,4*R*,5*R*)-3,4-Bis((*tert*-butyldimethylsilyl)oxy)-5-(6-chloro-9*H*-purin-9-yl)tetrahydrofuran-2-yl)methanol

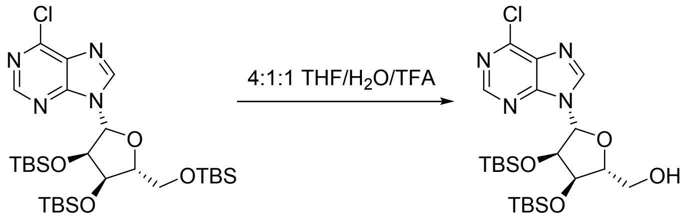

6

((2*R*,3*R*,4*R*,5*R*)-3,4-Bis((*tert*-butyldimethylsilyl)oxy)-5-(6-chloro-9*H*-purin-9-yl)tetrahydrofuran-2-yl)methanol was prepared by modification of a literature procedure.[3] *O*-TBS-protected 6-chloropurine **6** (1.07 g, 1.71 mmol) was dissolved in THF (17 mL) and cooled to 0 °C before a 1:1 mixture of TFA (4.25 mL) and H_2O (4.25 mL) was added and the reaction stirred at 0 °C for 5 hours. The reaction mixture was diluted to pH 7 with saturated aqueous $NaHCO_3$ solution and extracted with EtOAc. The organic layers were combined, washed with brine and dried over $MgSO_4$. The solvent was removed under reduced pressure and the crude product purified by column chromatography (4:1 cyclohexane/EtOAc) to give the title compound as a white solid (0.80 g, 91%); mp 153.3–154.4 °C (lit., 156–157 °C)[4]; δ_H (500 MHz, $CDCl_3$) 8.76 (s, 1H), 8.20 (s, 1H), 5.87 (d, *J* = 7.7 Hz, 1H), 4.96 (dd, *J* = 7.7, 4.5 Hz, 1H), 4.33 (d, *J* = 4.5 Hz, 1H), 4.20 – 416 (m, 1H), 3.94 (dd, *J* = 13.1, 1.9 Hz, 1H), 3.72 (dd, *J* = 13.1, 1.6 Hz, 1H), 0.94 (s, 9H), 0.72 (s, 9H), 0.12 (s, 3H), 0.11 (s, 3H), -0.14 (s, 3H), -0.66 (s, 3H); δ_C (126 MHz, $CDCl_3$) 152.6, 151.5, 150.6, 145.8, 133.7, 91.4, 89.6, 74.2, 73.8, 63.0, 25.9, 25.7, 18.2, 17.9, -4.4, -4.45, -4.49, -5.8; HRMS (ESI) $C_{22}H_{40}ClN_4O_4Si_2$ ($M+H^+$) requires 515.2271, found 515.2262.

9-((2*R*,3*R*,4*R*,5*R*)-3,4-Bis((tert-butyldimethylsilyl)oxy)-5-(((4-(trifluoromethyl)benzyl)oxy)methyl)tetrahydrofuran-2-yl)-6-chloro-9*H*-purine (Compound 7)

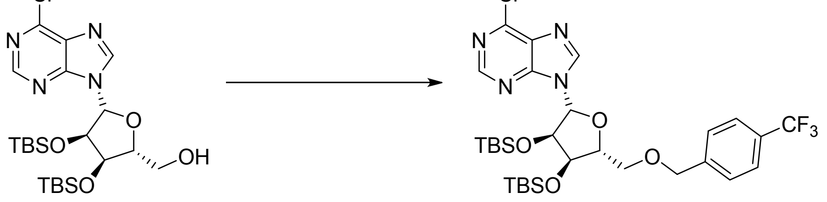

7

((2*R*,3*R*,4*R*,5*R*)-3,4-Bis((*tert*-butyldimethylsilyl)oxy)-5-(6-chloro-9*H*-purin-9-yl)tetrahydrofuran-2-yl)methanol (0.70 g, 1.36 mmol) was dissolved in DMF (12.5 mL) and cooled to -78 °C before the addition of NaH (60% dispersion in oil) (0.07 g, 1.77 mmol) and

[3] Zhu, X. F.; Williams, H. J.; Scott, A. I. *J. Chem. Soc., Perkin Trans. 1* **2000**, 2305.

the reaction stirred for 15 minutes. 1-(Bromomethyl)-4-(trifluoromethyl)benzene (0.65 g, 2.72 mmol) in DMF (5 mL) was added and the reaction allowed to slowly warm to room temperature over 16 hours. The reaction mixture was diluted with H_2O and extracted with EtOAc. The organic layers were combined, washed with brine and the solvent removed under reduced pressure. The crude product was purified by column chromatography (8:2 cyclohexane/EtOAc) to give compound **7** as a colourless oil (0.33 g, 53% based on recovered starting material); δ_H (500 MHz, $CDCl_3$) 8.73 (s, 1H), 8.57 (s, 1H), 7.64 (d, *J* = 8.0 Hz, 2H), 7.46 (d, *J* = 8.0 Hz, 2H), 6.08 (d, *J* = 3.8 Hz, 1H), 4.68 (s, 2H), 4.58 (app t, *J* = 3.9 Hz, 1H), 4.35 (dd, *J* = 5.2, 4.0 Hz, 1H), 4.30 – 4.23 (m, 1H), 3.92 (dd, *J* = 10.8, 3.1 Hz, 1H), 3.70 (dd, *J* = 10.8, 2.8 Hz, 1H), 0.90 (s, 9H), 0.83 (s, 9H), 0.06 (s, 3H), 0.05 (s, 3H), -0.01 (s, 3H), -0.10 (s, 3H); δ_C (126 MHz, $CDCl_3$) 152.1, 151.4, 151.2, 144.2, 141.4, 132.3, 130.5 (q, *J* = 32.3 Hz), 128.0, 125.8 (q, *J* = 3.8 Hz), 89.4, 83.6, 76.2*, 73.1, 71.6, 69.1, 25.9, 25.8, 18.2, 18.0, -4.2, -4.6, -4.7, -4.8; HRMS (ESI) $C_{30}H_{45}ClF_3N_4O_4Si_2$ (M+H$^+$) requires 674.2638, found 674.2655.

9-((2*R*,3*R*,4*R*,5*R*)-3,4-Bis((tert-butyldimethylsilyl)oxy)-5-(((4-(trifluoromethyl)benzyl)oxy)methyl)tetrahydrofuran-2-yl)-8-bromo-6-chloro-9*H*-purine (Compound 8)

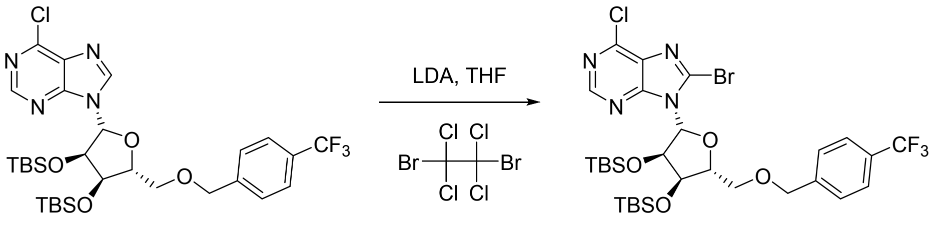

7 **8**

All glassware was dried in a 110 °C oven for 16 hours and THF was dried over 4 Å molecular sieves for 16 hours prior to use. Compound **7** (60 mg, 89 μmol) was dissolved in THF (1 mL) under N_2 and the solution cooled to -78 °C. LDA (2.0 M in THF) (63 μL, 125 μmol) was added dropwise at the reaction stirred for 35 minutes followed by the dropwise addition of 1,2-dibromotetrachloroethane (58 mg, 178 μmol) in THF (2 mL). The reaction was stirred at -78 °C for 90 minutes. The reaction mixture was then diluted with saturated aqueous NH_4Cl solution and the resulting mixture slowly warmed to room temperature and extracted with EtOAc. The organic extracts were combined, dried over $MgSO_4$ and the solvent removed under reduced pressure. The crude product was purified by column chromatography (10% EtOAc/cyclohexane) to give compound **8** as a colourless oil (62 mg, 92%); δ_H (500 MHz, $CDCl_3$) 8.60 (s, 1H), 7.56 (d, *J* = 8.1 Hz, 2H), 7.35 (d, *J* = 8.0 Hz, 2H), 6.05 (d, *J* = 5.2 Hz, 1H), 5.29 (dd, *J* = 5.3, 4.6 Hz, 1H), 4.65 – 4.58 (m, 2H), 4.54 (d, *J* = 12.6 Hz, 1H), 4.29 – 42.22 (m, 1H), 3.83 (dd, *J* = 10.5, 5.2 Hz, 1H), 3.72 (dd, *J* = 10.5, 5.6 Hz, 1H), 0.95 (s, 9H), 0.80 (s, 9H), 0.15 (s, 3H), 0.14 (s, 3H), -0.06 (s, 3H), -0.36 (s, 3H); δ_C (126 MHz, $CDCl_3$) 152.2, 151.6, 150.2, 142.1, 135.6, 132.6, 130.0 (q, *J* = 32.4 Hz), 127.4, 125.4 (q, *J* = 3.8 Hz), 91.4, 84.4, 72.71, 72.7, 72.5, 70.0, 26.0, 25.8, 18.2, 18.0, -4.2, -4.4, -4.5, -4.9; HRMS (ESI) $C_{30}H_{44}{}^{81}BrClF_3N_4O_4Si_2$ (M+H$^+$) requires 753.1729, found 753.1701.

9-((2*R*,3*R*,4*R*,5*R*)-3,4-Bis((tert-butyldimethylsilyl)oxy)-5-(((4-(trifluoromethyl)benzyl)oxy)methyl)tetrahydrofuran-2-yl)-6-chloro-*N*-(quinolin-6-ylmethyl)-9*H*-purin-8-amine (Compound 9)

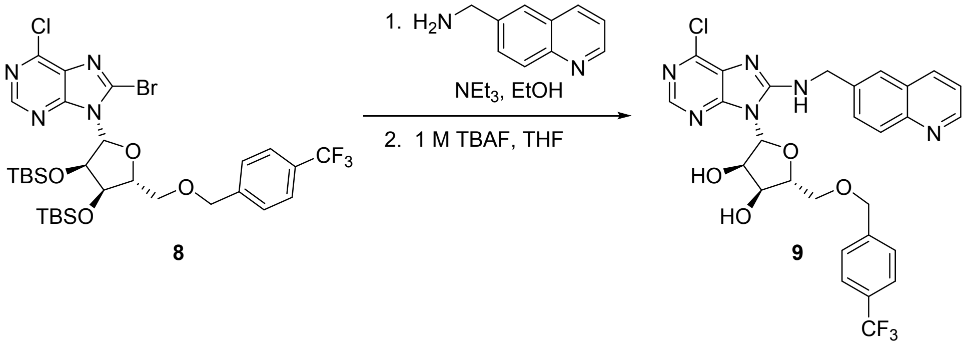

Compound **8** (90 mg, 0.12 mmol) was dissolved in THF (2.5 mL) and TBAF (1.0 M in THF, 3.1 eq) was added and the reaction stirred until complete by TLC analysis. The reaction mixture was diluted with MeOH, the solvent removed under reduced pressure to give the crude product. 63% of the isolated crude material was dissolved in EtOH (1.5 mL). Triethylamine (1.5 eq.) and quinolone-6-methyl amine (1.5 eq.) in EtOH (1.5 mL) were added and the reaction stirred at 40 °C for 16 hours under N_2. The solvent was removed under reduced pressure and the resulting solid taken up in CH_2Cl_2 and H_2O. The aqueous layer was extracted with CH_2Cl_2 and the organic layers combined, washed with brine and dried over $MgSO_4$. The solvent was removed under reduced pressure and the crude product purified by column chromatography (20:1 CH_2Cl_2/MeOH; R_f 0.33) to give compound **9** as a colourless oil (56 mg, 77%); δ_H (500 MHz, CD_3OD) 8.79 (dd, *J* = 4.3, 1.7 Hz, 1H), 8.35 (s, 1H), 8.27 – 8.21 (m, 1H), 7.98 (d, *J* = 8.7 Hz, 1H), 7.77 – 7.73 (m, 1H), 7.68 (dd, *J* = 8.8, 2.0 Hz, 1H), 7.48 (dd, *J* = 8.3, 4.3 Hz, 1H), 7.40 (d, *J* = 8.0 Hz, 2H), 7.20 (d, *J* = 8.0 Hz, 2H), 6.24 (d, *J* = 6.7 Hz, 1H), 4.90 (dd, *J* = 6.5, 5.7 Hz, 1H), 4.85 (obs d, *J* = 16.2 Hz, 1H), 4.69 (d, *J* = 16.2 Hz, 1H), 4.46 – 4.35 (m, 3H), 4.25 (app q, *J* = 2.4 Hz, 1H), 3.89 (dd, *J* = 10.8, 2.4 Hz, 1H), 3.71 (dd, *J* = 10.8, 2.4 Hz, 1H); δ_C (126 MHz, CD_3OD) 156.6, 154.8, 151.0, 149.3, 148.0, 143.12, 143.05, 138.6, 138.2, 132.4, 130.8 (q, *J* = 32.4 Hz), 130.4, 129.6, 129.4, 128.6, 126.5, 126.2 (q, *J* = 3.8 Hz), 122.8, 89.2, 85.8, 73.5, 72.9, 72.2, 71.2, 46.6; HRMS (ESI) $C_{28}H_{25}ClF_3N_6O_4$ (M+H$^+$) requires 601.1572, found 601.1557.

(2*R*,3*R*,4*S*,5*R*)-2-(6-((6-Aminohexyl)amino)-8-((quinolin-6-ylmethyl)amino)-9*H*-purin-9-yl)-5-(((4-(trifluoromethyl)benzyl)oxy) methyl)tetrahydrofuran-3,4-diol

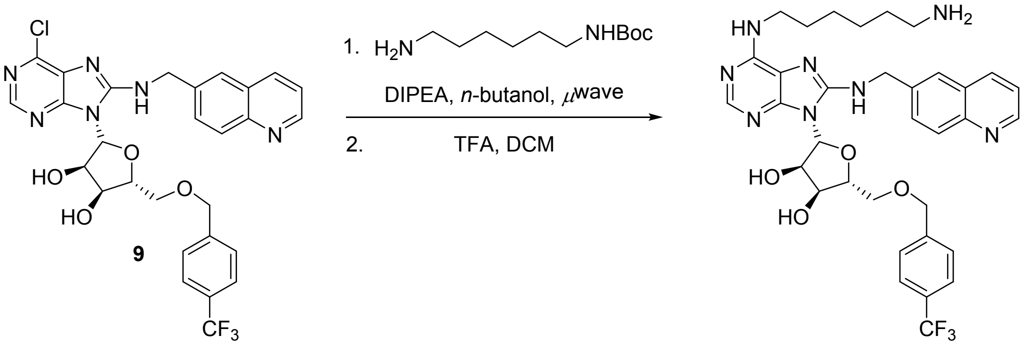

N-Boc-1,6-hexanediamine (0.10 g, 0.47 mmol) and DIPEA (20 μL, 0.11 mmol) were added compound **9** (56 mg, 0.09 mmol) in *n*-butanol (0.20 mL) and the reaction heated in the μwave for 2 hours at 120 °C. The crude product was filtered through a plug of silica to remove excess *N*-boc-1,6-hexanediamine. The filtrate was concentrated under reduced pressure and 50% of the recovered material dissolved in CH_2Cl_2 (0.50 mL). TFA (0.25 mL) was added dropwise and the reaction stirred at room

temperature for 2 hours. The solvent was removed under reduced pressure and the crude product purified by semi-preparative HPLC (method A) to give the title compound as a pale yellow oil (12 mg, 33%); δ_H (500 MHz, $(CD_3)_2SO$) 8.84 (dd, *J* = 4.2, 1.6 Hz, 1H), 8.38 (br s, 2H), 8.24 (d, *J* = 8.4 Hz, 1H), 7.98 – 7.92 (m, 2H), 7.82-7.78 (m, 1H), 7.69 (dd, *J* = 8.7, 1.8 Hz, 1H), 7.54 (d, *J* = 8.1 Hz, 2H), 7.47 (dd, *J* = 8.3, 4.2 Hz, 1H), 7.36 (d, *J* = 8.0 Hz, 2H), 7.26 (t, *J* = 5.9 Hz, 1H), 6.87 (t, *J* = 6.0 Hz, 1H), 5.94 (d, *J* = 5.7 Hz, 1H), 4.96 (app t, *J* = 5.6 Hz, 1H), 4.71 – 4.47 (m, 4H), 4.36 – 4.30 (m, 1H), 4.09 – 4.02 (m, 1H), 3.77 (dd, *J* = 10.7, 3.0 Hz, 1H), 3.65 (dd, *J* = 10.8, 4.4 Hz, 1H), 3.47 – 3.35 (m, 2H), 2.76 – 2.62 (m, 2H), 1.59 – 1.41 (m, 4H), 1.27 – 1.16 (m, 4H); δ_C (126 MHz, $(CD_3)_2SO$) 151.6, 151.2, 150.1, 149.0, 147.0, 142.8, 138.1, 135.6, 129.4, 129.1, 128.9, 128.8, 127.91 (q, *J* = 30.7 Hz), 127.6, 125.1, 125.0 (q, *J* = 3.8 Hz), 121.5, 121.4, 87.0, 82.9, 71.5, 70.6, 70.4, 70.34, 45.3, 40.2, 38.7, 27.3, 26.0, 25.7; HRMS (ESI) $C_{34}H_{40}F_3N_8O_4$ (M+H$^+$) requires 681.3119, found 681.3109.

Bisaryl-FAM (10)

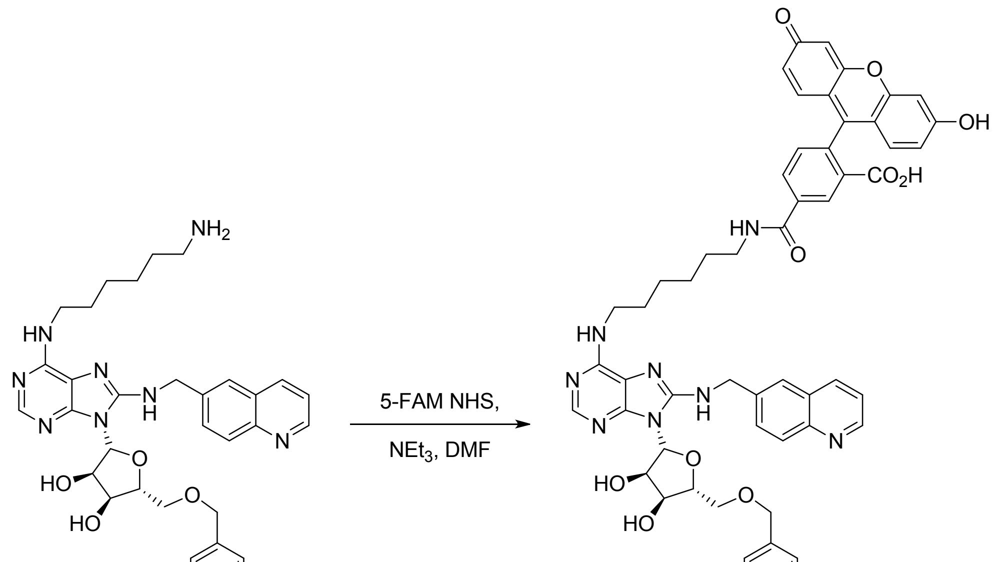

bisaryl-FAM 10

Triethylamine (1 µL, 9 µmol) and 5-carboxyfluorescein NHS ester (5 mg, 11 µmol) were added to a solution of (2*R*,3*R*,4*S*,5*R*)-2-(6-((6-aminohexyl)amino)-8-((quinolin-6-ylmethyl)amino)-9*H*-purin-9-yl)-5-(((4-(trifluoromethyl)benzyl)oxy)methyl) tetrahydrofuran-3,4-diol (6 mg, 9 µmol) in DMF (1 mL) and the reaction stirred at room temp for 6 hours. The reaction mixture was concentrated under reduced pressure and the crude product purified by semi-preparative HPLC (method C) to give bisaryl-FAM as an orange oil (4 mg, 42%); δ_H (500 MHz, CD_3OD) 8.77 (dd, *J* = 4.3, 1.8 Hz, 1H), 8.43 – 8.38 (m, 1H), 8.22 (d, *J* = 7.7 Hz, 1H), 8.13 (dd, *J* = 8.0, 1.6 Hz, 1H), 8.03 (s, 1H), 7.95 (d, *J* = 8.7 Hz, 1H), 7.68 (d, *J* = 2.0 Hz, 1H), 7.61 (dd, *J* = 8.7, 1.9 Hz, 1H), 7.47 (dd, *J* = 8.3, 4.3 Hz, 1H), 7.34 (d, *J* = 8.1 Hz, 2H), 7.27 (d, *J* = 8.1 Hz, 1H), 7.25 (d, *J* = 8.1 Hz, 2H), 6.69 (d, *J* = 2.3 Hz, 2H), 6.63 (d, *J* = 8.6 Hz, 2H), 6.54 (dd, *J* = 8.7, 2.4 Hz, 2H), 6.16 (d, *J* = 6.9 Hz, 1H), 4.85 – 4.82 (obs dd, *J* = 6.7, 5.9 Hz 1H), 4.61 (d, *J* = 16.5 Hz, 1H), 4.52 (d, *J* = 16.4 Hz, 1H), 4.50 – 4.40 (m, 3H), 4.24 (app q, *J* = 2.5 Hz, 1H), 3.92 (dd, *J* = 10.8, 2.5 Hz, 1H), 3.75 (dd, *J* = 10.8, 2.2 Hz, 1H), 3.51 – 3.44 (m, 2H), 3.38 (app t, *J* = 7.0 Hz, 2H), 1.67 – 1.56 (m, 4H), 1.47 – 1.37 (m, 4H); δ_C (126 MHz, MeOH) 168.4, 153.1, 152.7, 151.0, 150.7, 148.0, 143.1, 139.1, 138.2, 137.9, 135.8, 130.8 (q, *J* = 31.9 Hz), 130.42, 130.2, 129.8, 129.4, 128.8, 126.26 (q, *J* = 3.8 Hz), 125.9, 125.5*, 124.7*, 122.8, 117.8, 114.0*, 103.7, 88.7, 85.6, 73.7, 72.8, 72.2, 71.4, 46.6, 41.6, 41.11, 30.6, 30.3, 27.8, 27.6[4]; HRMS (ESI) $C_{55}H_{51}F_3N_8O_{10}$ $(M+2H)^{2+}$ requires 520.1835, found 520.1837.

[4] Several quaternary carbons not observed in spectra

Bisaryl-ATTO-488 (11)

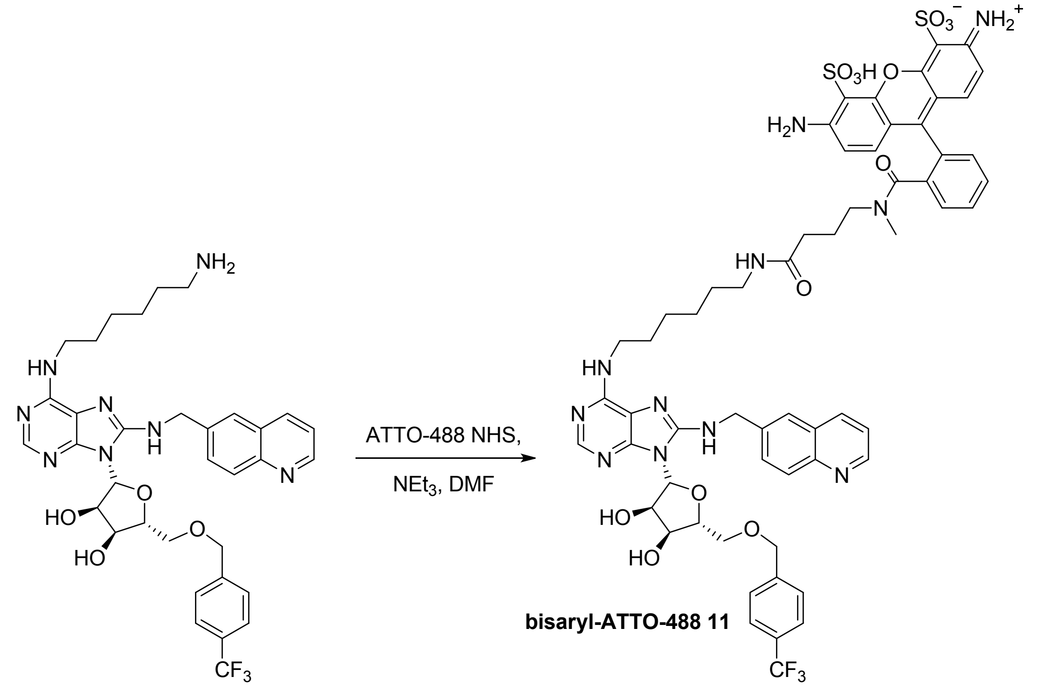

bisaryl-ATTO-488 11

Triethylamine (1 µl, 9 µmol) and ATTO488 NHS ester (6 mg, 9 µmol) were added to a solution of (2*R*,3*R*,4*S*,5*R*)-2-(6-((6-aminohexyl)amino)-8-((quinolin-6-ylmethyl)amino)-9*H*-purin-9-yl)-5-(((4-(trifluoromethyl)benzyl)oxy)methyl)tetrahydrofuran-3,4-diol (6 mg, 9 µmol) in DMF and the reaction stirred at room temperature for 6 hours. The solvent was removed under reduced pressure and the crude product purified by semi-preparative HPLC (method A) to give bisaryl-ATTO-488 as an orange oil (4 mg, 34%); δ_H (500 MHz, $(CD_3)_2SO$) δ 9.20 – 9.12 (m, 1H), 8.83 – 8.78 (m, 1H), 8.49 (br s, 1H), 8.23 (d, *J* = 7.4 Hz, 1H), 7.94 (d, *J* = 8.9 Hz, 2H), 7.80 (br s, 1H), 7.72 – 7.66 (m, 2H), 7.54 (d, *J* = 8.1 Hz, 2H), 7.50 – 7.40 (m, 2H), 7.36 (d, *J* = 8.0 Hz, 2H), 7.04 (d, *J* = 9.5 Hz, 2H), 6.97 (dd, *J* = 9.3, 3.5 Hz, 2H), 5.92 (d, *J* = 5.6 Hz, 1H), 5.02 – 4.92 (m, 1H), 4.73 – 4.60 (m, 2H), 4.50 (s, 2H), 4.36 – 4.28 (m, 1H), 4.03 (obs, 1H), 3.77 (dd, *J* = 10.8, 3.1 Hz, 1H), 3.64 (dd, *J* = 10.8, 4.4 Hz, 1H), 3.08 (t, *J* = 7.0 Hz, 2H), 2.93 (t, *J* = 6.9 Hz, 2H), 2.08 (s, 3H), 1.97 (t, *J* = 7.3 Hz, 1H), 1.75 (t, *J* = 7.1 Hz, 1H), 1.65 – 1.10 (m, 12H)[5]; δ_C (126 MHz, $(CD_3)_2SO$) 155.3, 150.1, 149.0, 147.0, 138.1, 135.7, 131.5, 129.3, 129.2, 128.9, 125.2, 125.0, 121.5, 118.7, 113.1, 87.0, 82.8, 71.9, 71.5, 70.6, 70.3, 46.0, 45.3, 30.7, 29.1, 26.24, 26.19, 25.2, 22.0[6]; HRMS (ESI) $C_{59}H_{62}F_3N_{11}O_{13}S_2$ $(M+2H)^{2+}$ requires 626.6956, found 626.6971.

(2*R*,3*R*,4*S*,5*R*)-2-(6-Amino-8-((quinolin-6-ylmethyl)amino)-9*H*-purin-9-yl)-5-(((4(trifluoromethyl)benzyl)oxy)methyl) tetrahydrofuran-3,4-diol (Compound 2)

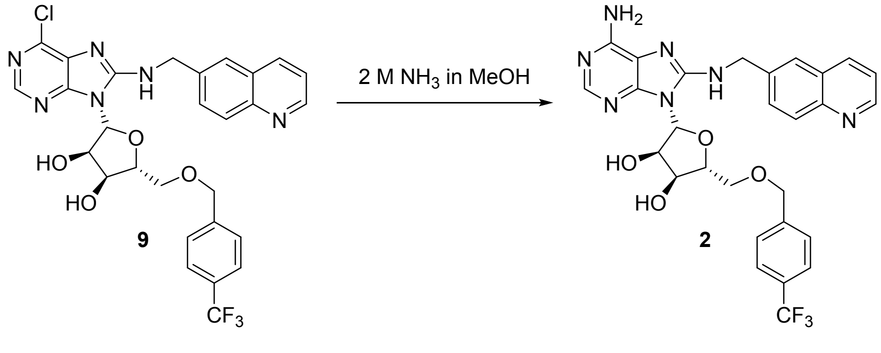

[5] Minor impurities present in spectra, exchangeable protons not observed owing to prior solution in CD_3OD.
[6] Several carbons not observed in spectra

Compound **9** (30 mg, 50 μmol) was dissolved in 2.0 M NH_3 in MeOH (1.5 mL) and the reaction heated in the *μ*wave to 150 °C for 12 hours. The solvent was removed under reduced pressure and the product purified by semi-preparative HPLC (method A) to give compound **2** as a colourless oil (13 mg, 45%); δ_H (500 MHz, CD_3OD) 8.79 (dd, *J* = 4.3, 1.7 Hz, 1H), 8.25 – 8.21 (m, 1H), 8.00 (s, 1H), 7.97 (d, *J* = 8.7 Hz, 1H), 7.71 – 7.67 (m, 1H), 7.62 (dd, *J* = 8.8, 2.0 Hz, 1H), 7.48 (dd, *J* = 8.3, 4.3 Hz, 1H), 7.35 (d, *J* = 8.1 Hz, 2H), 7.25 (d, *J* = 8.0 Hz, 2H), 6.18 (d, *J* = 6.9 Hz, 1H), 4.86 – 4.82 (m, 1H), 4.63 (d, *J* = 16.5 Hz, 1H), 4.53 (d, *J* = 16.5 Hz, 1H), 4.50 – 4.40 (m, 3H), 4.25 (app q, *J* = 2.3 Hz, 1H), 3.93 (dd, *J* = 10.7, 2.4 Hz, 1H), 3.75 (dd, *J* = 10.8, 2.2 Hz, 1H); δ_C (126 MHz, CD_3OD) 153.6, 153.1, 151.4, 151.0, 150.4, 148.0, 143.1, 139.0, 138.2, 130.8 (q, *J* = 32.4 Hz), 130.2, 129.8, 129.4, 128.8, 126.3 (q, *J* = 3.8 Hz), 125.9, 122.8, 117.6, 88.7, 85.6, 73.7, 72.8, 72.2, 71.4, 46.5; HRMS (ESI) $C_{28}H_{27}F_3N_7O_4$ (M+H$^+$) requires 585.2071, found 582.2051.

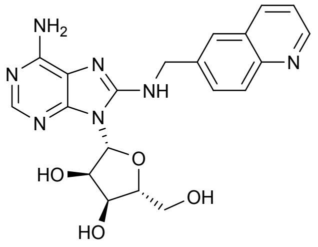

Quinoline **12** ((2*R*,3*R*,4*S*,5*R*)-2-(6-amino-8-((quinolin-6-ylmethyl)amino)-9*H*-purin-9-yl)-5-(hydroxymethyl)tetrahydrofuran-3,4-diol) was synthesised in 1 step from 8-bromoadenosine using the method previously described by Cheeseman et al.[7]

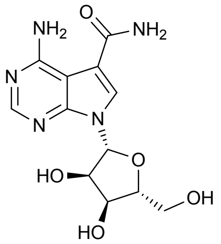

Sangivamycin **13** was purchased from Sigma-Aldrich and used without further purification http://www.sigmaaldrich.com/catalog/product/sigma/s5895?lang=en&region=GB (17/11/16)

[7] M. D. Cheeseman, I. M. Westwood, O. Barbeau, M. Rowlands, S. Dobson, A. M. Jones, F. Jeganathan, R. Burke, N. Kadi, P. Workman, I. Collins, R. L. M. van Montfort, K. Jones, *J. Med. Chem.*, 2016, **59**, 4625-4636.

NMR-Spectra of key compounds

Compound 7

^{1}H NMR ($CDCl_3$)

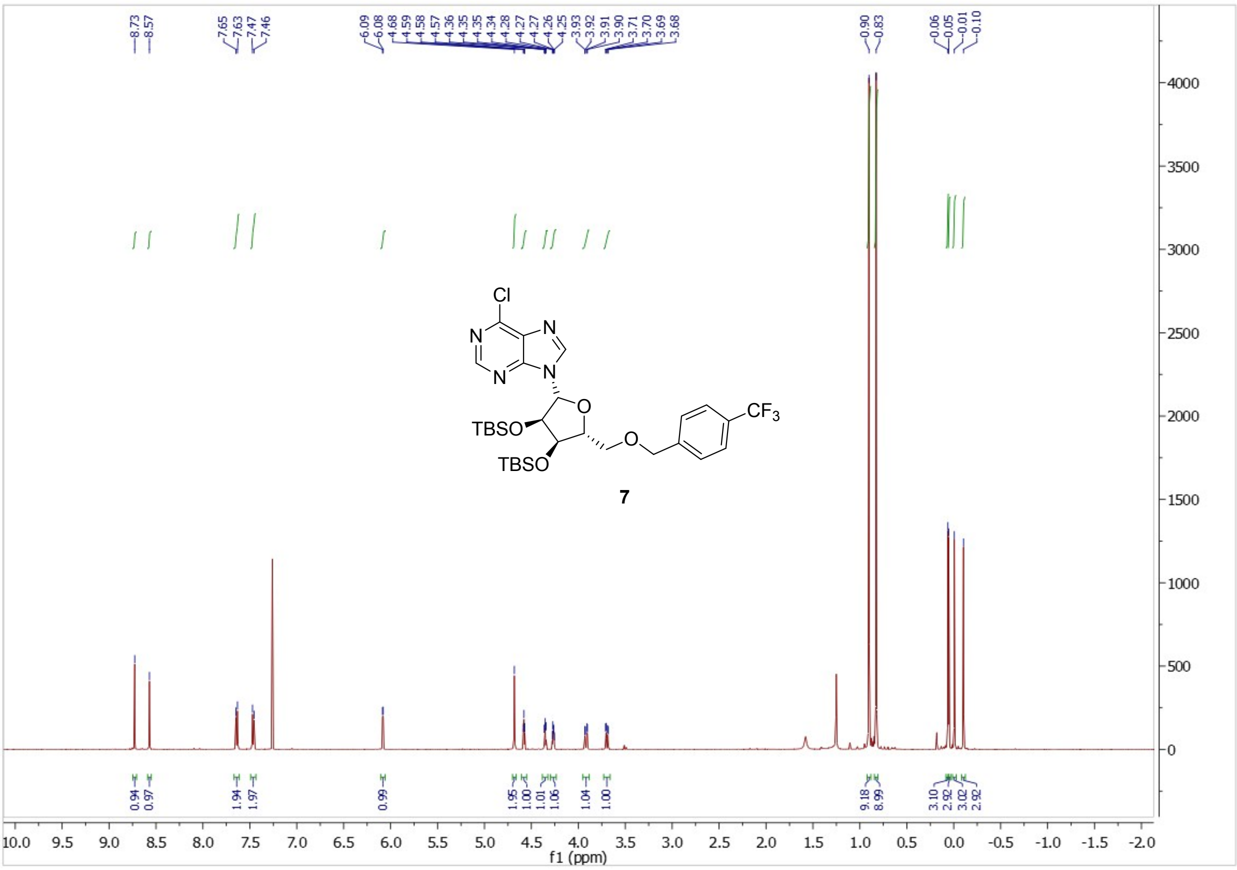

^{13}C NMR ($CDCl_3$)

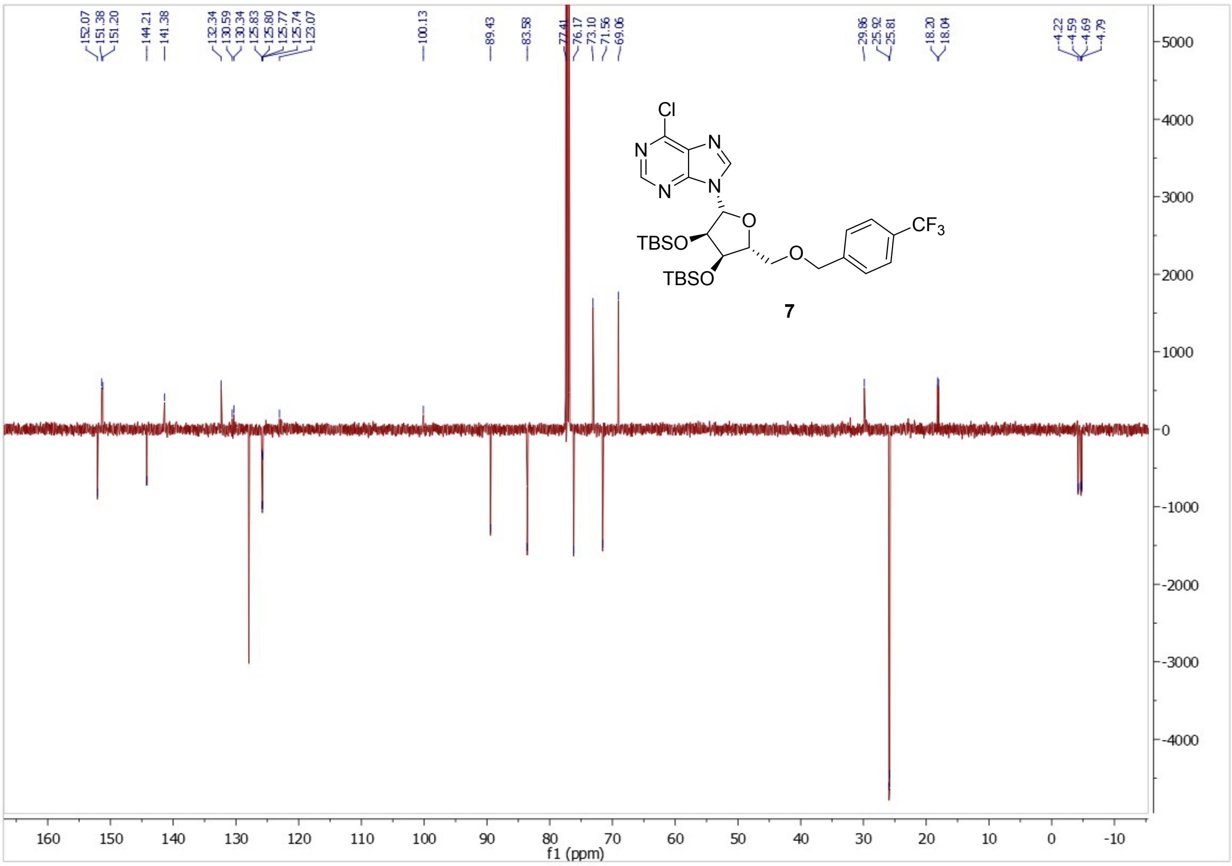

Compound 8

^{1}H NMR ($CDCl_3$)

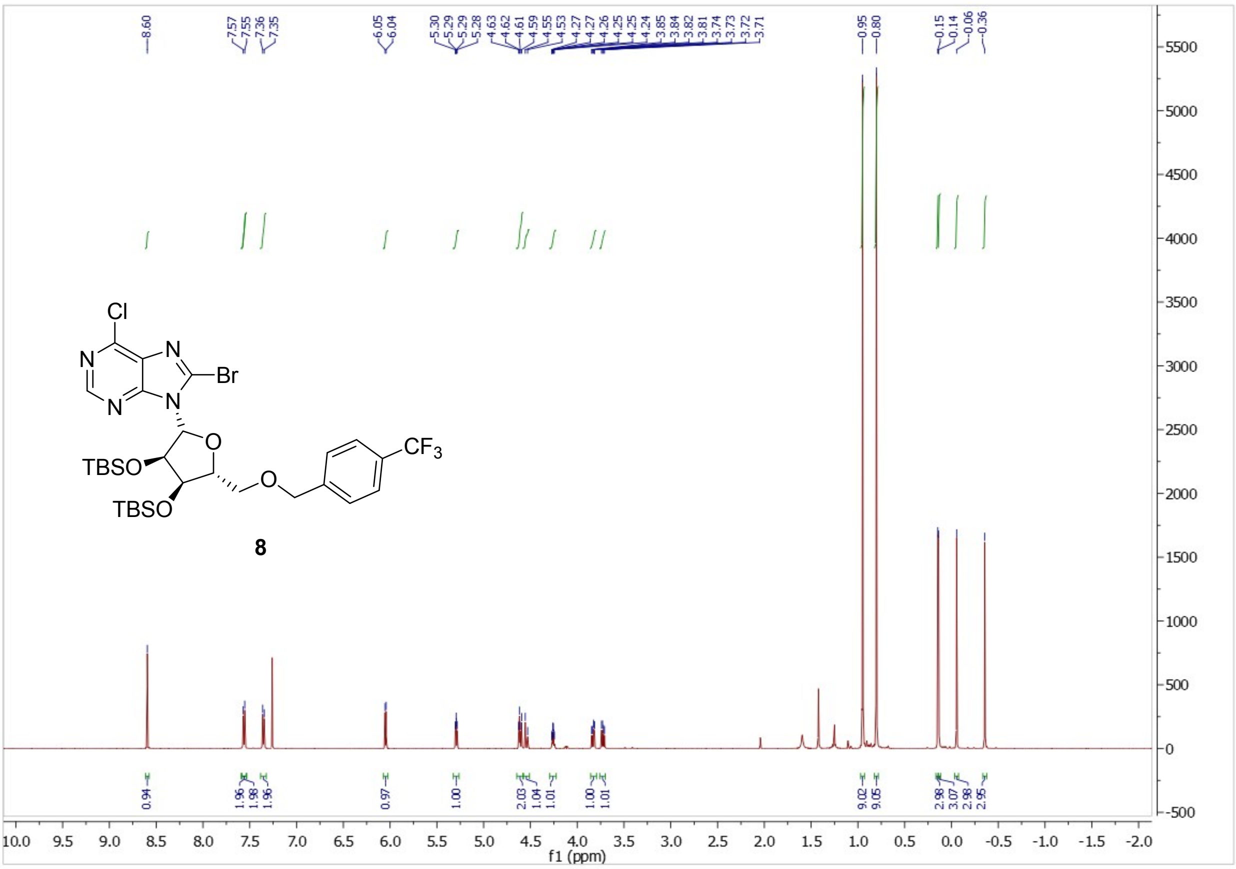

^{13}C NMR ($CDCl_3$)

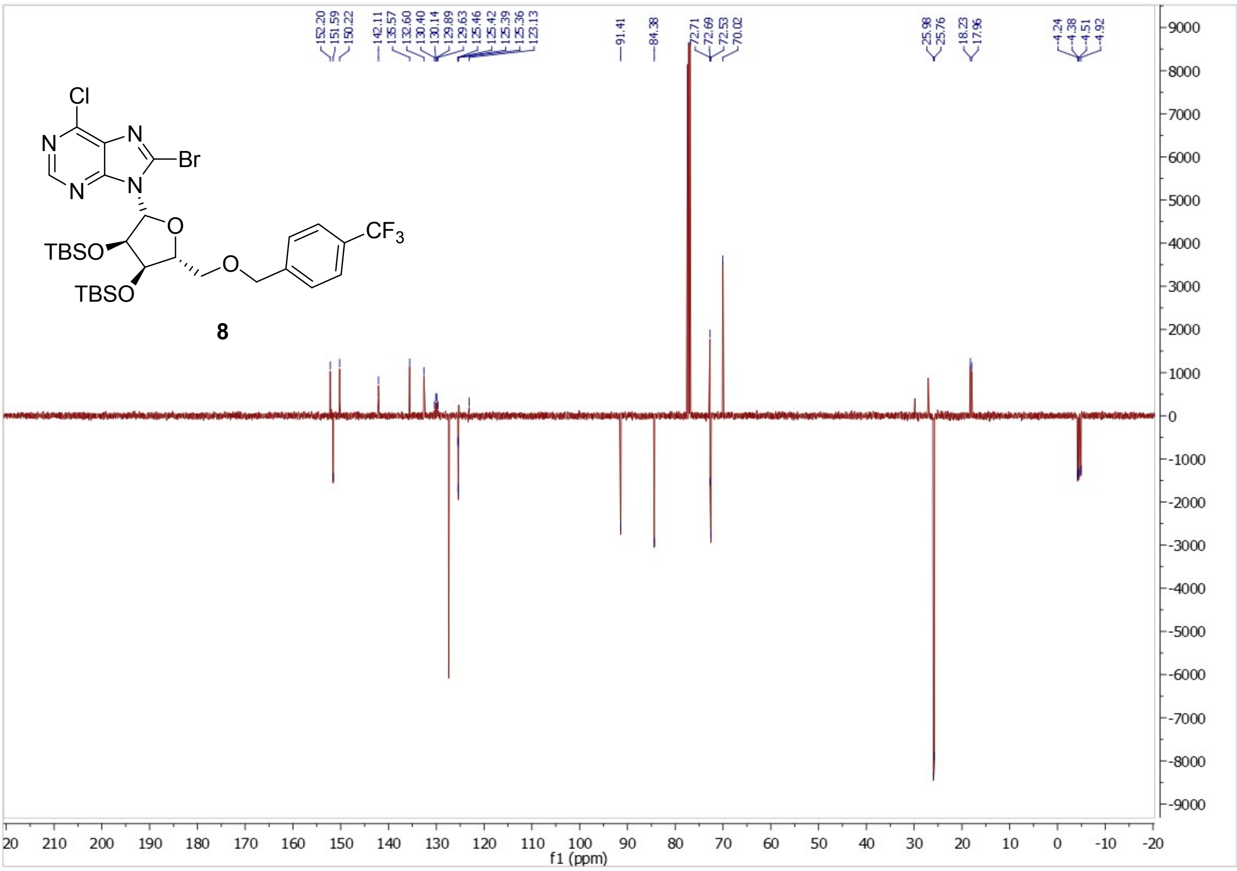

Compound 9

^{1}H NMR (CD_3OD)

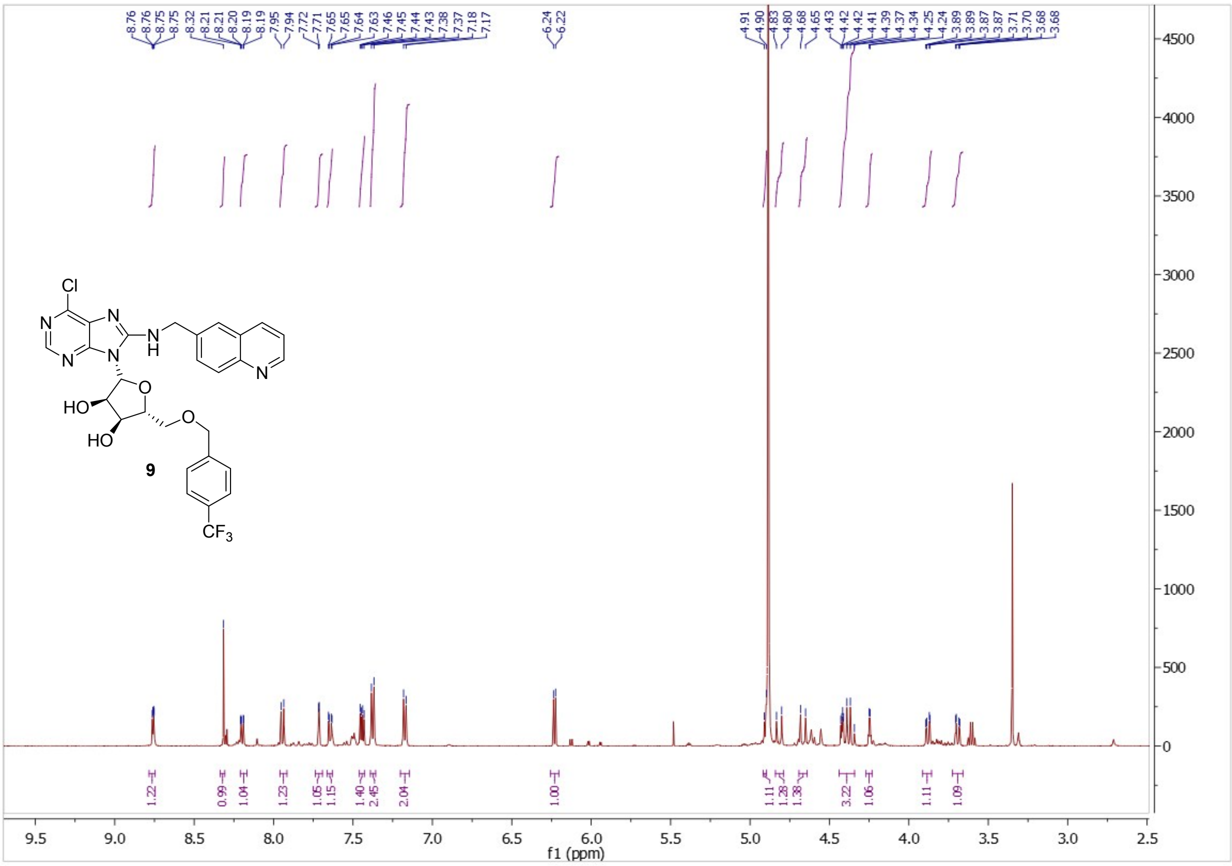

^{13}C NMR (CD_3OD)

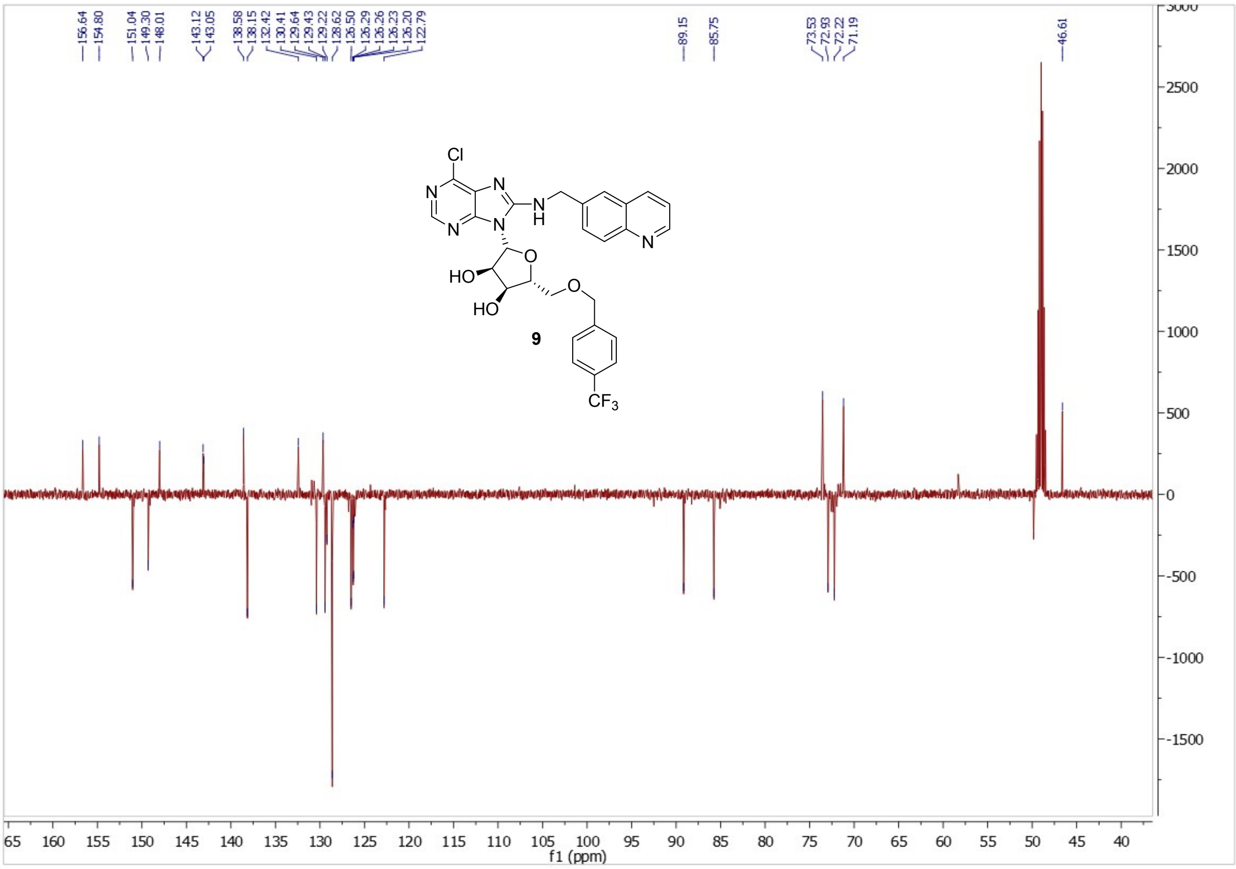

Compound 11

^{1}H NMR

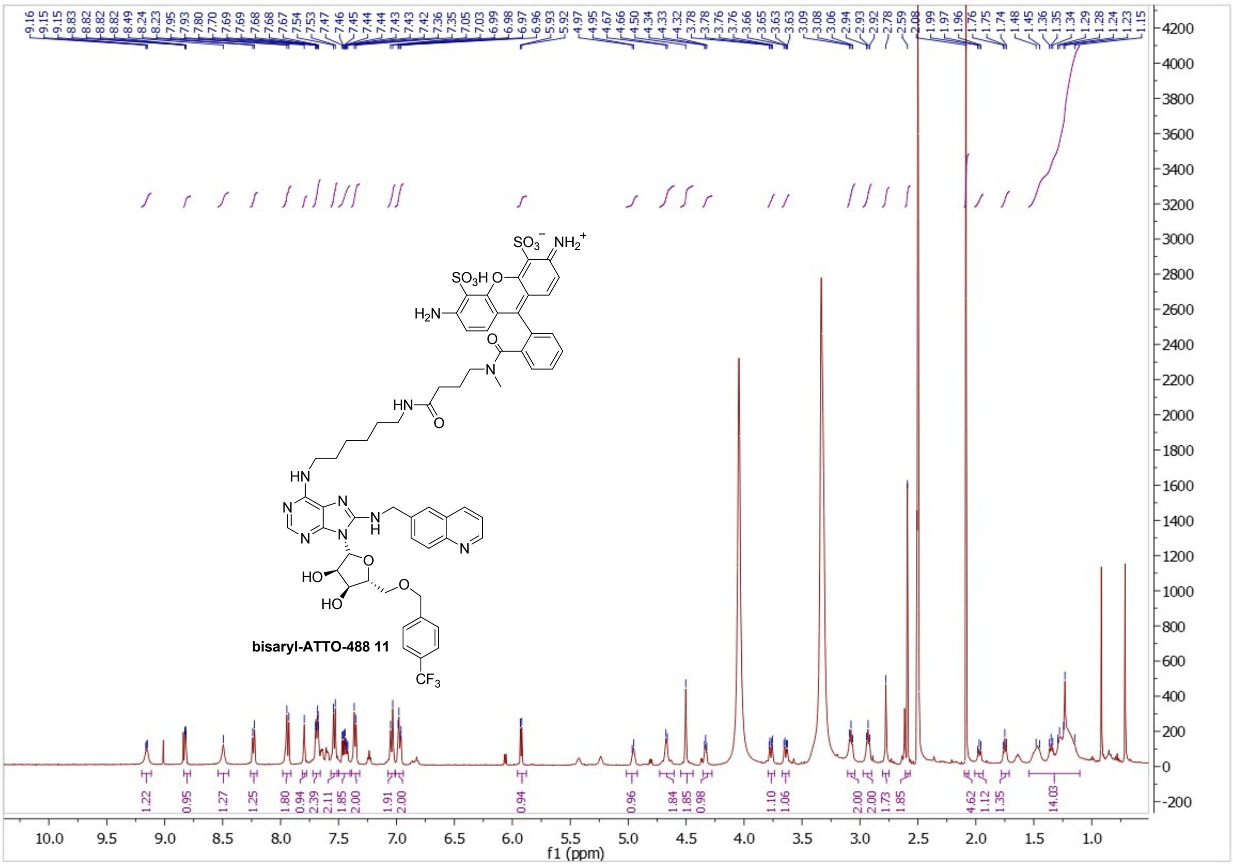

^{13}C NMR

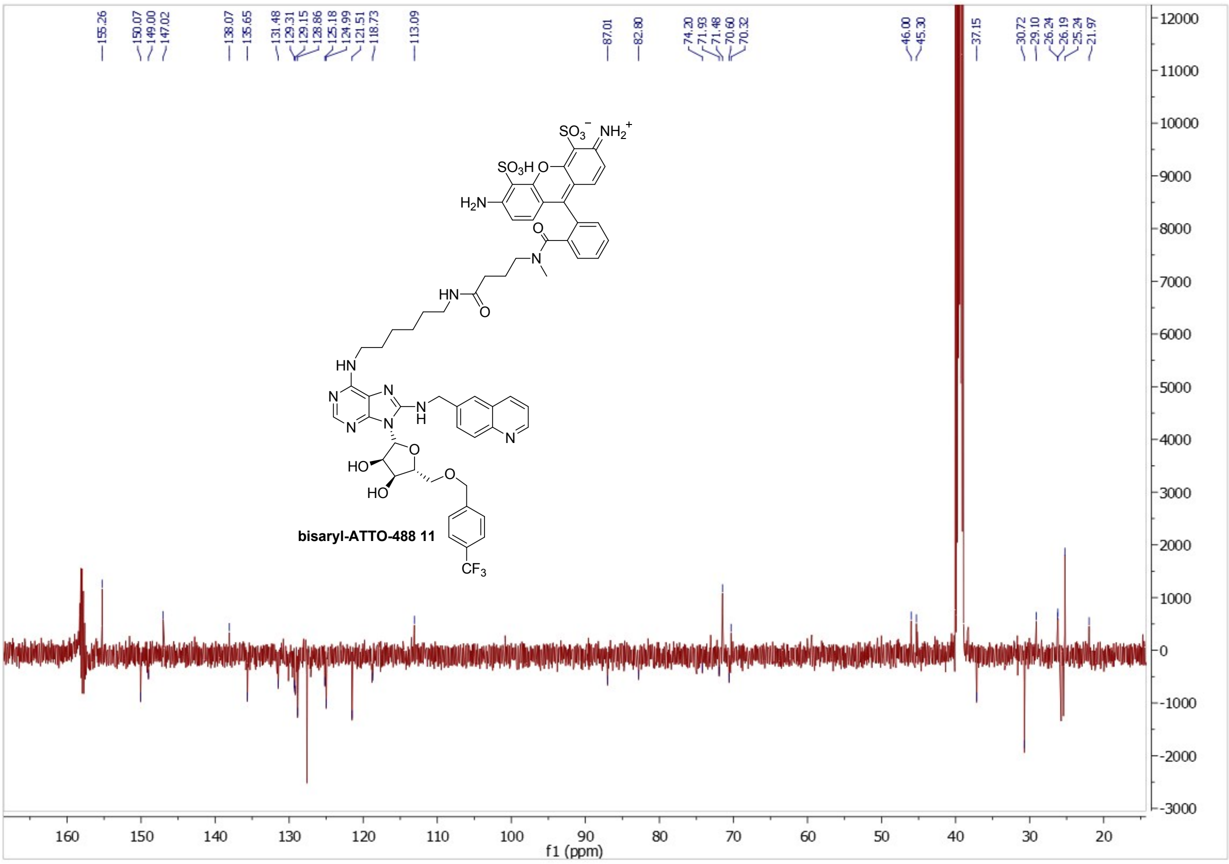

HSP70 Crystallography Analysis

All crystal structures were analysed using MOE 2017.09

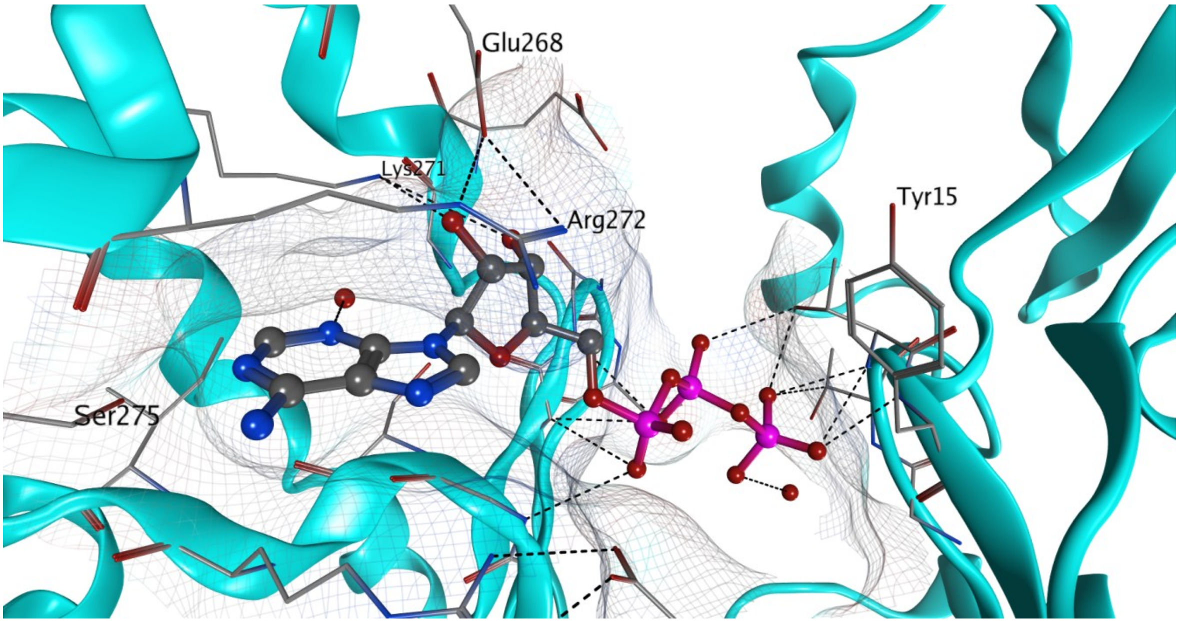

Figure S8. Cartoon representation describing the key binding interactions of ATP bound to HSC70-NBD/tr-BAG1 (PDB: 3ATU), blue=nitrogen, grey=carbon, red=oxygen, pink=phosphate, hydrogen bonds are represented by black dashes.

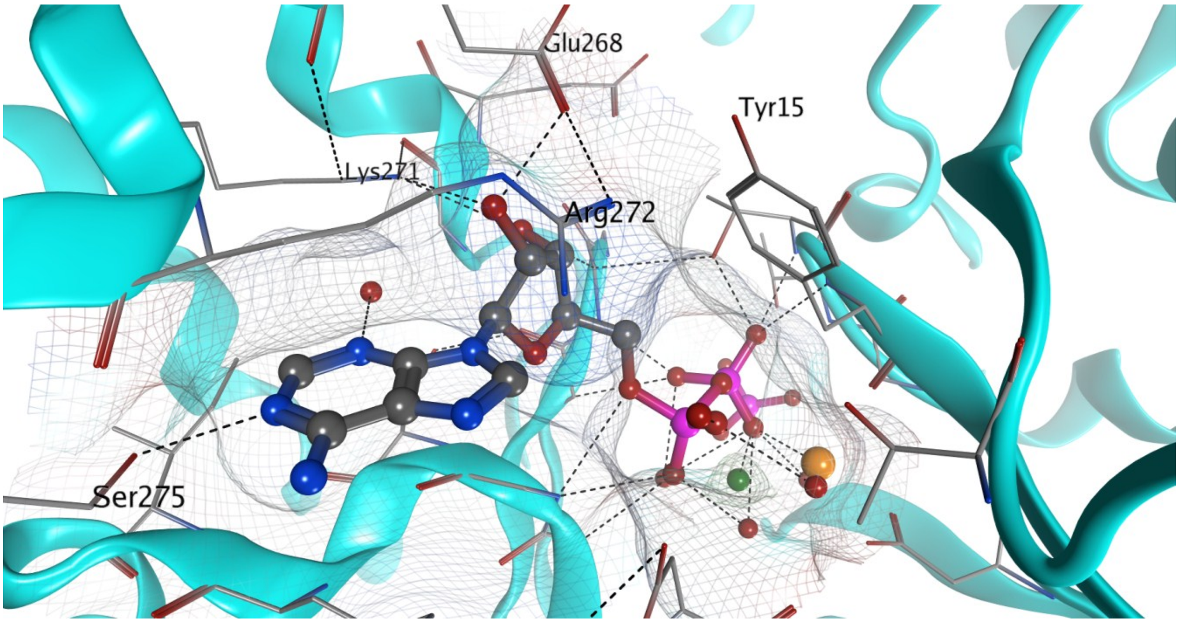

Figure S9. Cartoon representation describing the key binding interactions of ADP/Pi bound to HSP72-NBD (PDB: 3ATU), blue=nitrogen, grey=carbon, red=oxygen, pink=phosphate, green=magnesium, orange=sodium, hydrogen bonds are represented by black dashes.

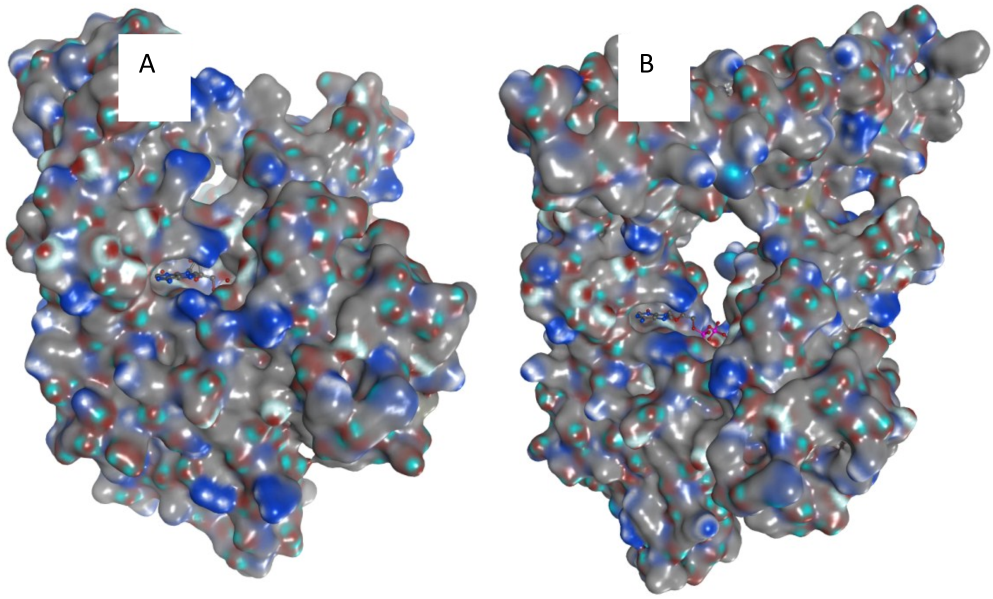

Figure S10. Protein surface representations of nucleotides bound to HSP70. A) ADP/Pi bound to HSP72-NBD. ADP/Pi induces the closed conformation of HSP72-NBD (PDB: 3ATU), the protein wraps around the ligand, requiring a protein conformational change in order for the ADP/Pi ligand to dissociate. B) tr-BAG1 induces an apparent open conformation of HSC70-NBD (PDB: 3FZF), leaving a large gap in the centre of the protein.

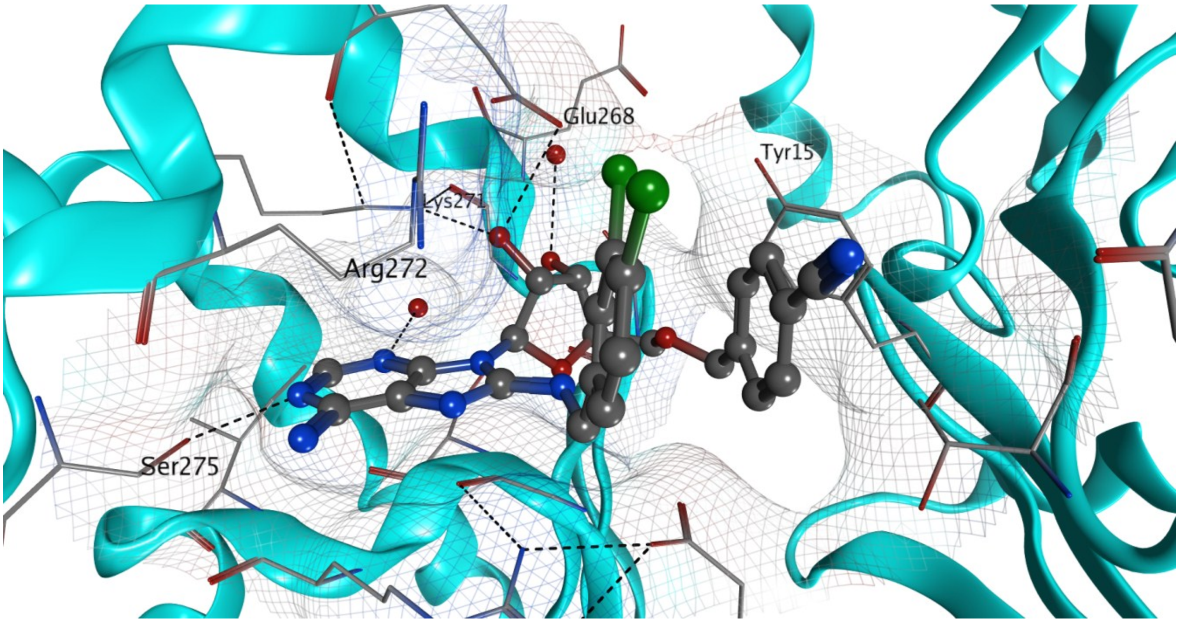

Figure S11. Cartoon representation describing the key binding interactions of Ver-155008 **1** bound to HSP72-NBD/tr-BAG1 (PDB: 4IO8), blue=nitrogen, grey=carbon, red=oxygen, pink=phosphate, hydrogen bonds are represented by black dashes. Ver-155008 1 binds in an apparent intermediate conformation of the NBD.

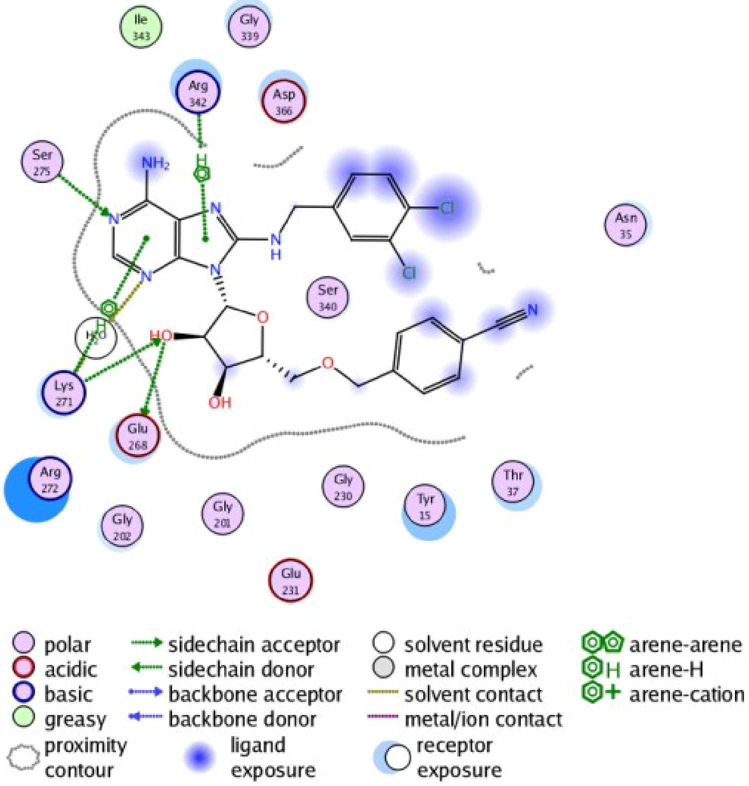

Figure S12. Ligand interaction analysis of Ver-155008 **1** bound to HSP72-NBD. The *N*6-adenine position was solvent exposed so was selected as the point for fluorophore attachment.

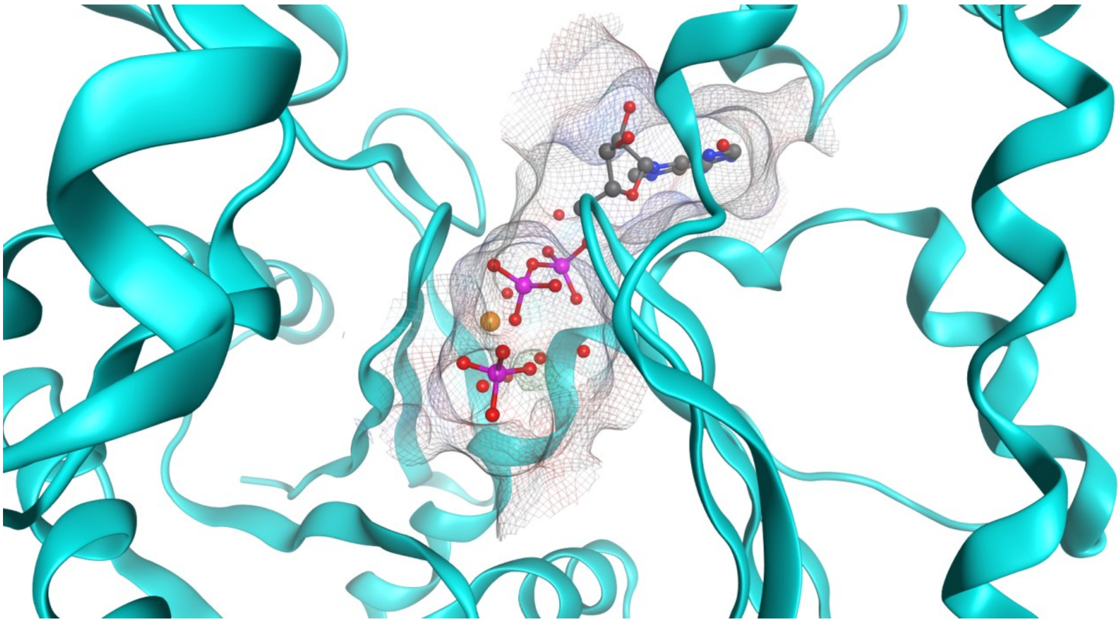

Figure S13. The phosphate binding pocket of ADP/P_i bound to HSP72-NBD. Our data is consistent with BAG1 causing a conformational change of the PBP to facilitate first P_i dissociation, followed by ADP. Because BAG1 does not inhibit ligands binding to the adenine binding pocket, we hypothesised that the HSP72/BAG1 complex could represent a more druggable protein target than the HSP72 protein alone.
